# Postnatal state transition of cardiomyocyte as a primary step in heart maturation

**DOI:** 10.1007/s13238-022-00908-4

**Published:** 2022-04-08

**Authors:** Zheng Li, Fang Yao, Peng Yu, Dandan Li, Mingzhi Zhang, Lin Mao, Xiaomeng Shen, Zongna Ren, Li Wang, Bingying Zhou

**Affiliations:** 1 Shenzhen Key Laboratory of Cardiovascular Disease, Fuwai Hospital Chinese Academy of Medical Sciences, Shenzhen, 518057 China; 2 State Key Laboratory of Cardiovascular Disease, Fuwai Hospital, National Center for Cardiovascular Diseases, Chinese Academy of Medical Sciences and Peking Union Medical College, Beijing, 100037 China; 3 Key Laboratory of Pluripotent Stem Cells in Cardiac Repair and Regeneration, Chinese Academy of Medical Sciences, Beijing, 100037 China; 4 State Key Laboratory of Cardiovascular Disease, Fuwai Hospital, National Center for Cardiovascular Diseases, Chinese Academy of Medical Sciences and Peking Union Medical College, Beijing, 100037 People’s Republic of China

**Keywords:** heart maturation, state transition, cardiomyocyte, JUN, heart regeneration

## Abstract

Postnatal heart maturation is the basis of normal cardiac function and provides critical insights into heart repair and regenerative medicine. While static snapshots of the maturing heart have provided much insight into its molecular signatures, few key events during postnatal cardiomyocyte maturation have been uncovered. Here, we report that cardiomyocytes (CMs) experience epigenetic and transcriptional decline of cardiac gene expression immediately after birth, leading to a transition state of CMs at postnatal day 7 (P7) that was essential for CM subtype specification during heart maturation. Large-scale single-cell analysis and genetic lineage tracing confirm the presence of transition state CMs at P7 bridging immature state and mature states. Silencing of key transcription factor JUN in P1-hearts significantly repressed CM transition, resulting in perturbed CM subtype proportions and reduced cardiac function in mature hearts. In addition, transplantation of P7-CMs into infarcted hearts exhibited cardiac repair potential superior to P1-CMs. Collectively, our data uncover CM state transition as a key event in postnatal heart maturation, which not only provides insights into molecular foundations of heart maturation, but also opens an avenue for manipulation of cardiomyocyte fate in disease and regenerative medicine.

**Supplementary Information:**

The online version contains supplementary material available at 10.1007/s13238-022-00908-4.

## INTRODUCTION

Functional maturation with concomitant, near complete loss of proliferative potential, are hallmarks of mature cardiomyocytes. Understanding the mechanisms underlying heart maturation is not only essential to filling our knowledge gaps of cardiac biology, but also paves the way for artificial manipulation of cardiomyocyte fate in cardiac disease intervention and regeneration.

At the cellular level, cardiac maturation is generally considered a process where cardiomyocytes undergo a series of maturational changes, including the transition from hyperplastic to hypertrophic growth, cell cycle arrest, sarcomeric protein isoform switching, and a shift from glycolysis to oxidative phosphorylation. Early gene expression studies directly compared the fetal or neonatal heart to their adult counterpart as a means to elucidate mechanisms of cardiac maturation (Mahdavi et al., 1982; Eulalio et al., 2012; Gilsbach et al., 2014, 2018; Morikawa et al., 2017; Mohamed et al., 2018). However, the discovery of postnatal regenerative capacity during the first week (i.e., postnatal day (P) 1–7) of murine life (Porrello et al., 2011) suggested a rather complicated picture of cardiac maturation. Studies have since attempted to explore the defining characteristics of this regenerative time window, uncovering transcription factor Meis1 (Mahmoud et al., 2013), the oxygen-rich postnatal environment (Puente et al., 2014) and thyroid hormones (Hirose et al., 2019) as important inducers of cell cycle exit in cardiomyocytes. However, little is known about the molecular determinants and the biological implications of this unique time window. Hence, there is still a substantial gap between our knowledge of regulatory factors that control cardiomyocyte maturation and the innate molecular changes that take place within cardiac cells, especially cardiomyocytes, during *in vivo* maturation*.*

RNA sequencing and its high-resolution counterpart, single-cell RNA-sequencing (scRNA-seq), have greatly facilitated our understanding of postnatal heart development (Hu et al., 2018). Curiously, none of the current transcriptional studies included the “miraculous” postnatal day 7 (Gan et al., 2015; Sim et al., 2015; DeLaughter et al., 2016; Hu et al., 2018; Talman et al., 2018). Despite that, the use of scRNA-seq in studying postnatal cardiomyocytes was hampered by the fact that mature cardiomyocytes are rod-shaped, reaching an average of ~100 μm along the longitudinal axis. Taking advantage of an established single-cell analysis platform suitable for mature cardiomyocytes (Yao et al., 2018; Ren et al., 2020; Wang et al., 2020a, 2020b), as well as genetic animal models, we made the unexpected observation of a transition state of cardiomyocytes at postnatal day 7, manifested as transcriptional and epigenetic reduction of cardiac attributes, that is essential for physiological cardiac maturation, and demonstrates potential in cardiac repair.

## RESULTS

### Transcriptomic and epigenetic decline of cardiac gene expression at postnatal day 7

**Figure 1:**
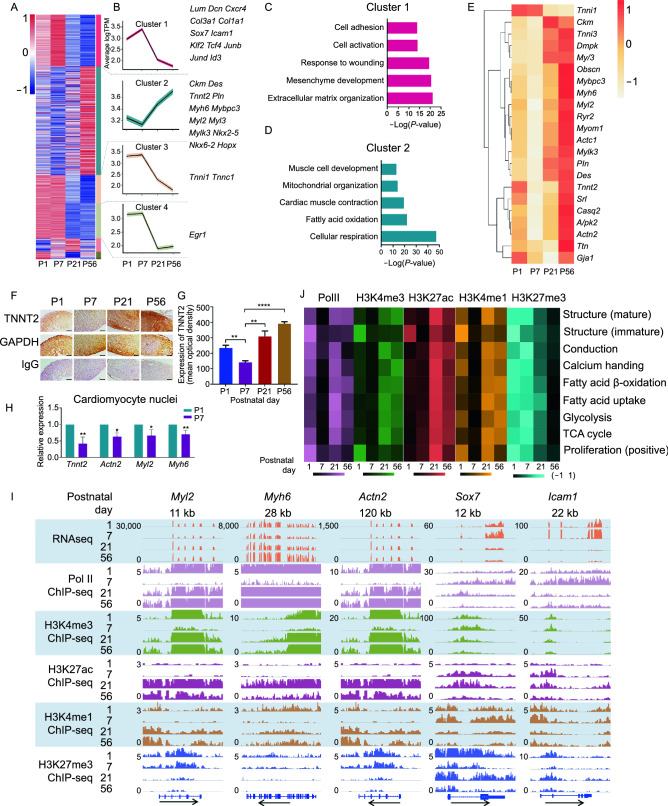
**Transcriptional and epigenetic decline of cardiac gene expression at postnatal day 7**. (A) Hierarchical clustering of differentially expressed genes (DEGs) in cardiomyocytes (CMs) isolated from hearts on postnatal days (P) 1, 7, 21, and 56, respectively. Isolated cardiomyocytes were subjected to bulk RNA-seq. Data shown are from two independent biological replicates. (B) The mean (black) and confidence interval of a standard *t*-test (colored) of genes is plotted over time to show the expression patterns of Clusters 1–4 as determined in (A). (C and D) Gene ontology (GO) analyses of Clusters 1 (C) and 2 (D) show the enrichment of biological processes of respective genes. Selected top categories are shown. Please see Table S1 for the full list. (E) Heatmap displaying expression of a subset of cardiac genes in cardiomyocytes across indicated time points. (F) Expression of cardiac markers TNNT2 and of house-keeping protein GAPDH in consecutive heart sections at indicated time points measured by immunohistochemical staining. IgG serves as a negative control. Scale bar = 50 μm. (G) Quantification of protein expression in (F). Mean optical density is calculated as the integrated optical density (IOD) divided by the distribution area of the target protein. The *P* values were calculated by one-way ANOVA, then by post hoc analysis. *n* = 4, ***P < 0.001. (H) Real-time PCR to show the expression of cardiac genes in PCM1^+^ cardiomyocyte nuclei isolated from P1 versus P7 hearts. The values were plotted as Mean ± SEM from 3 independent experiments. *P* values were calculated by Student’s *t*-test, *P < 0.05, **P < 0.01. (I) Genome tracks showing RNA-Seq, polymerase II ChIP-Seq, H3K4me3 ChIP-Seq, H3K27ac ChIP-Seq, H3K27ac ChIP-Seq, H3K4me1 ChIP-Seq, and H3K27me3 ChIP-Seq at *Myl2*, *Myh6*, *Actn2*, *Sox7*, and *Icam1* gene loci, respectively, in cardiomyocytes isolated from indicated time points. (J) Heatmap showing enrichment of polymerase II and histone modifications within 5 kb of the transcription start sites in selected gene categories. The shades of color indicate median expression for each category of genes. Please see Table S2 for the full list

To gain an overview of the transcriptional events during mammalian cardiac maturation, with particular interest in P7, we first performed RNA sequencing (RNA-seq) on freshly isolated cardiomyocytes (CMs) from mouse hearts at postnatal days 1 (P1), 7 (P7), 21 (P21) and 56 (P56). Quality control of isolated cardiomyocytes was performed to ensure the viability and purity of isolated cells (Fig. S1A–C). Clustering of differentially expressed genes (DEGs) revealed 4 major expression patterns (Clusters 1–4) (Fig. 1A and 1B). Cluster 1, whose average expression peaked at P7, and dropped abruptly thereafter, included many extracellular matrix genes and non-cardiac transcription factors, such as *Lum*, *Dcn*, *Klf2* and *Tcf4*. By contrast, cluster 2, which almost displayed the opposite expression (lowest at P7, followed by steady increase) encompassed nearly all well-known cardiac-specific structural genes and transcription factors, including *Tnnt2*, *Myh6*, and *Nkx2-5* (Fig. 1B). Gene Ontology enrichment analysis predicted that genes in Cluster 1 were involved in cell-microenvironment interactions, whereas genes in Cluster 2 were related to metabolic and contractile elements of the heart (Fig. 1C and 1D; Table S1). Closer inspection of the expression of well-known structure- and contraction-related genes demonstrated *bona fide* reduction at P7 (Fig. 1E). We further examined the expression of proliferation and cell-cycle related genes (Fig. S1D and S1E). Surprisingly, contrary to our common perception that cardiomyocyte lose proliferative capacity by P7, we observed that the majority of genes analyzed (96 out of 109, 88.07%) had similar expression levels between P1 and P7, which sharply dropped by D21. The majority (88 out of 96, 91.67%) of genes with this pattern of expression (i.e., P1 and P7 high) had even slightly higher expression at P7 compared to P1, indicating that P7-CMs were not significantly different from P1-CMs in terms of the expression of proliferation-related genes. To rule out potential influence of the isolation procedure and non-cardiomyocyte contamination on gene expression, and to examine whether such changes can be observed at the protein level, we examined cardiac gene expression *in situ* using immunohistochemistry in heart sections from the same time points. Quantification of staining intensity confirmed that the normalized expression of TNNT2 protein was lowest at P7, a pattern that mirrored its transcriptional changes (Fig. 1F and 1G). We further isolated cell nuclei directly from P1 and P7 hearts, which were subsequently sorted for CM nuclei (PCM1^+^) by fluorescence-activated cell sorting (FACS). In line with previous results, the expression of representative cardiac genes (*Tnnt2*, *Actn2*, *Myl2* and *Myh6*) was reduced in P7 cardiomyocyte nuclei compared to their P1 counterparts (Fig. 1H). Similar patterns of cardiac gene expression in postnatal hearts were observed in 2 additional mouse strains and 2 rat strains, indicating conservation of this postnatal mechanism (Fig. S2).

Epigenetic regulation, especially histone modifications, often play critical roles in defining cell state, by activating, repressing or poising gene expression (Paige et al., 2012; Wamstad et al., 2012). Therefore, we profiled H3K4me3, H3K27me3, H3K4me1, H3K27ac, and polymerase II (Pol II) binding across the genome of CMs at indicated time points. Instead of establishing a poised chromatin state, active histone marker H3K4me3 at cardiac gene loci (e.g., *Myl2*, *Myh6*, and *Actn2*) almost diminished at P7, consistent with our observations at the transcription level (Figs. 1I and S3). Likewise, both H3K27ac and H3K4me1, histone markers for active and potential enhancers, were markedly reduced at cardiac genes at P7 (Fig. 1I). On the contrary, apparent increase in active histone modifications, as well as removal of repressive histone markers (H3K27me3), were observed at genes from Cluster 1, such as *Sox7* and *Icam1*, both of which displayed elevated expression in RNA analysis (Fig. 1I). Further, polymerase II occupancy on these genes closely reflected their gene expression at P7 (Fig. 1I). Globally, histone markers and polymerase II binding on genes characteristic of cardiomyocyte structure and function demonstrated similar dynamics (Fig. 1J; Table S2). Collectively, these findings demonstrated both transcriptomic and epigenetic reduction of cardiac gene expression at P7, suggesting a transient, but molecularly distinct, process in cardiomyocyte development after birth.

### CMs assume a transcriptionally distinct state at P7 with transiently elevated expression of non-cardiac transcription factors

**Figure 2:**
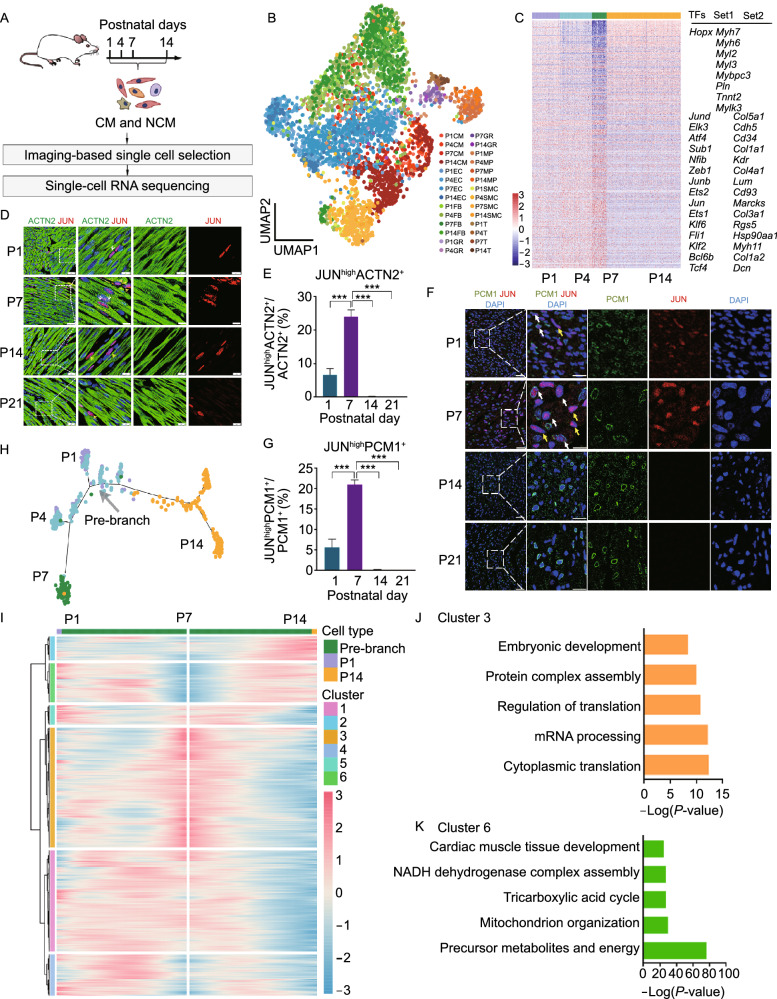
**CMs assume a transcriptionally distinct state at P7 with transiently elevated expression of non-cardiac transcription factors**. (A) Schematic of study design. Cardiac cells were isolated from postnatal mice on P1, P4, P7, and P14, and subjected to scRNA-seq. CM, cardiomyocyte; NCM, non-cardiomyocyte. (B) Uniform Manifold Approximation and Projection (UMAP) to display clustering of single cells isolated from hearts at indicated developmental stages. CM, cardiomyocyte; EC, endothelial cell; FB, fibroblast; GR, granulocyte; MP, macrophage; SMC, smooth muscle cell; T, T cell. (C) Heatmap showing the differentially expressed transcription factors (TFs), cardiac genes (Set 1), and P7-abundant genes (Set 2) in CMs at indicated time points as determined by single-cell RNA-Seq. Please see Table S4 for the full list. (D) Immunofluorescent (IF) staining against ACTN2 and JUN in heart sections at indicated time points. White arrows indicate co-localized cells, yellow arrows represent cells with JUN staining only. Scale bar = 25 μm in lower magnification, = 10 μm in higher magnification. (E) Quantitative analysis of JUN^high^ACTN2^+^ cells (D) in heart sections at indicated time points. *P* values were calculated by one-way ANOVA, then by post hoc analysis. *n* = 3, ***P < 0.001. (F) Immunofluorescent (IF) staining against CM nucleus marker PCM1 and JUN in heart sections at indicated time points. White arrows indicate co-localized cells, yellow arrows represent cells with JUN staining only. Scale bar = 25 μm in lower magnification, = 10 μm in higher magnification. (G) Quantitative analysis of JUN^high^PCM1^+^ cells (F) in heart sections at indicated time points. *P* values were calculated by one-way ANOVA, then by post hoc analysis. *n* = 3, ***P < 0.001. (H) Monocle analysis showing the ordering of CMs along pseudotime. Each point represents a single CM, while each color indicates a time point. (I) Heatmap to show differentially expressed genes along pseudotime in Monocle analysis. Please see Table S5 for the full list. (J and K) Gene ontology (GO) analyses of Clusters 1 (J) and 2 (K) show the enrichment of biological processes of respective genes. Selected top categories are shown. Please see Table S6 for the full list

To further confirm gene expression alterations and scrutinize the developmental trajectory of CMs after birth, we performed single-cell RNA-sequencing (scRNA-seq) on cells isolated from hearts at P1, P4, P7 and P14 after birth (Figs. 2A and S4A). A total of 5,010 single cells from multiple anatomic regions were analyzed using a well-established scRNA-seq platform (Kim et al., 2018; Yao et al., 2018), where visual inspection of stained cells prior to selection precludes cell aggregates and dead cells (Fig. S4A and S4B). Following strict quality control, 4,231 cells were subjected to subsequent analyses, with a median depth of 206,606 reads/cell, 84% alignment rate/cell, and 2,899 genes/cell (Fig. S4B and S4C). To classify cells in the heart at each time point, we performed clustering analysis with Seurat, and defined major cell clusters, including cardiomyocyte (CM), endothelial cell (EC), fibroblast (FB), macrophage (MP), and smooth muscle cell (SMC) clusters, based on their specific molecular markers (Figs. 2B and S4D). Possible batch effects among animals were ruled out by the relatively even expression of pan-house-keeping genes in every single cell, as well as by closely clustered cell populations using these genes (Fig. S4E and S4F; Table S3). In line with bulk RNA-seq data, expression of representative cardiac genes (Set 1), transiently decreased, while extracellular matrix genes were upregulated at P7 (Set 2) (Fig. 2C). Given a predominant role of transcription factors in defining cell identity (Lee and Young, 2013), we further determined transcription factors that were differentially expressed in CMs at P7 versus all other time points. More than 100 transcription factors exhibited significantly different expression levels in P7-CMs compared to other time ponts (Fig. 2C; Table S4). Surprisingly, factors implicated in stemness (e.g., *Klf2* and *Tcf4*) exhibited a marked increase at P7, suggesting a transcriptionally unsettled state in CMs (Fig. 2C).

To verify the physical presence of these special CMs during postnatal heart development, we first performed immunofluorescent (IF) staining against ACTN2 (CM marker) and JUN (one of top-ranked markers) at various developmental stages. Strikingly, the percentage JUN^high^-CMs was 6.67% at P1, reached a peak level of 24.00% at P7, and sharply declined to 0.18% and 0.07% at days P14 and P21, respectively (Fig. 2D and 2E). Similar results were also obtained via co-staining of JUN and PCM1 (Fig. 2F and 2G). Stemness marker Krüppel-like factor 2 (KLF2) exhibited the same trends as JUN (Fig. S4H–K). Together, these findings suggested substantial presence of this unique CM state at P7.

Next, we aligned CMs of different postnatal days along pseudotime using Monocle to characterize their developmental status. P7-CMs and P14-CMs were situated at two distinct ends of the trajectory, indicating large transcriptional differences between them (Fig. 2H). Similarly, a similar transcriptional status was also uncovered using Slingshot and diffusion map, respectively, where P7 CMs do not link P4 and P14 CMs (Fig. S4L and S4M). Gene Ontology analysis of DEGs along the developmental trajectory in Monocle revealed that genes highly expressed in P7-CMs (Cluster 3) were enriched in functions related to mRNA processing and protein synthesis, whereas those highly expressed in P1-CMs and P14-CMs (Cluster 6) were related to cardiac metabolism and cardiac muscle tissue development (Fig. 2I–K; Tables S5 and S6). These observations indicated a non-unidirectional maturation path of CMs where P7-CMs exhibit attributes of a transition state that may be prerequisite for further fate commitment.

### Transcriptomic cell state transition of CMs at P7 during postnatal CM maturation

**Figure 3:**
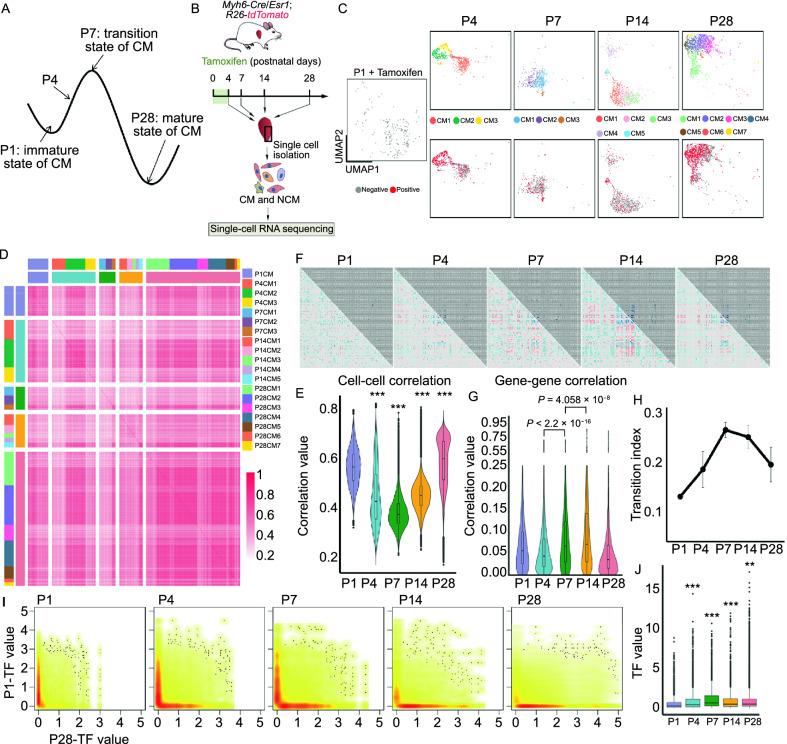
**Transcriptomic cell state transition of CMs at P7 during postnatal CM maturation**. (A) Schematic to illustrate the proposed model that P1-CM needs to destabilize P1 state to achieve a transition state at P7, and then specify into mature states (P28). (B) Workflow of scRNA-seq experiment. *Myh6*-*Cre*/*Esr1*;*R26*-*tdTomato* mice were treated with tamoxifen between P1–P4. Cells were collected from P4, P7, P14, and P28 hearts, and then subjected to scRNA-seq. *n* = 3 biological replicates for each time point. (C) UMAP showing the distribution of tdTomato^+^ cells in each CM subtype at each developmental stage. Top: CM subtypes; bottom: tdTomato^+^ cells (red) versus tdTomato^−^ cells (grey). Please see Table S7 for the full list. (D) Cell-cell correlation matrices showing correlation coefficient of all pairs of CMs at indicated time points. (E) Average correlation coefficients for cell-cell pairs at indicated time points (D). (F) Gene-gene correlation matrices for all genes at indicated time points. Blue color represents negative correlation, red color indicates positive correlation. (G) Average correlation coefficients for gene-gene pairs at indicated time points. (H) State transition indices at various time points. (I) Expression levels of P1-CM- and P28-CM-specific transcription factors in CMs at indicated time points. (J) Quantification of (I)

The above observations let us to propose a model in which CMs need to work against the stability of perinatal gene expression configuration immediately after birth, before triggering the postnatal development program to gain molecular and functional maturation (Fig. 3A). In this regard, P7-CMs should represent a critical transition state between the neonatal state (P1) and states of fully mature CMs (P28). To test this hypothesis, we performed single-cell RNA-Seq using *Myh6-Cre*/*Esr1*;*R26*-*tdTomato* transgenic mice (Fig. 3B). This animal model permanently labels CMs with tdTomato (RFP) upon treatment with tamoxifen, and thus offers the opportunity to exclude non-CM cell types from downstream analysis. Following stringent quality control, a total of 11,168 cells were used in subsequent analyses (Fig. S5A). We achieved a median depth of 148,398 reads/cell, 81% alignment rate/cell, and 1,461 genes/cell (Fig. S5B–D). Upon projection of cells by Uniform Manifold Approximation and Projection (UMAP), we visualized individual CM subtypes, as well as RFP-positive CMs during postnatal heart development (Figs. S5E–H and 3C; Table S7). Notably, RFP^+^-CMs were present in all CM subtypes at all stages, supporting the idea that *Myh6*-RFP^+^ CMs at P1 gave rise to all CM subtypes (Fig. 3C). Taking it a step further, we calculated cell-cell correlation and gene-gene correlation of 1,687 CMs from all 19 CM states spread over 5 time points (1 at P1, 3 at P4, 3 at P7, 5 at P14, and 7 at P28) using a previously reported algorithm (Mojtahedi et al., 2016). Interestingly, cell-cell correlation decreased from P1 to P7, reaching a lowest point at P7, and then increased from P7 to P28 (Fig. 3D and 3E). This finding reflected increased amplitudes of random fluctuations in gene expression at P7 due to the weakening attracting force of P1 state prior to postnatal maturation. By contrast, gene-gene correlation steadily increased from P4 to P14, and then declined at P28, indicating an increase of correlation coefficients between all pairs of genes required for symmetry-breaking destabilization in a state (Fig. 3F and 3G). Calculation of the transition index *I*_C_, a ratio of gene-gene correlation to cell-cell correlation, unveiled a peak at P7, suggesting a critical transition state at P7 during postnatal maturation of CMs (Fig. 3H). As TF-centered gene regulatory network (GRN) defines a stable state, we next determined the expression of representative TFs of both P1 and P28 in every single CM at each time point. Compared to relative co-expression TFs at P1 and P28, TFs in P7-CMs displayed bifurcation patterns in their expression, suggesting stochastic fluctuations in gene regulatory networks driving transitions (P7) between coexisting states (P1, P28) (Fig. 3I and 3J). Collectively, these observations supported the idea that postnatal CMs need to pass a transition at P7 to continue towards final maturation.

### Potential involvement of P7-CM in CM subtype specification

**Figure 4:**
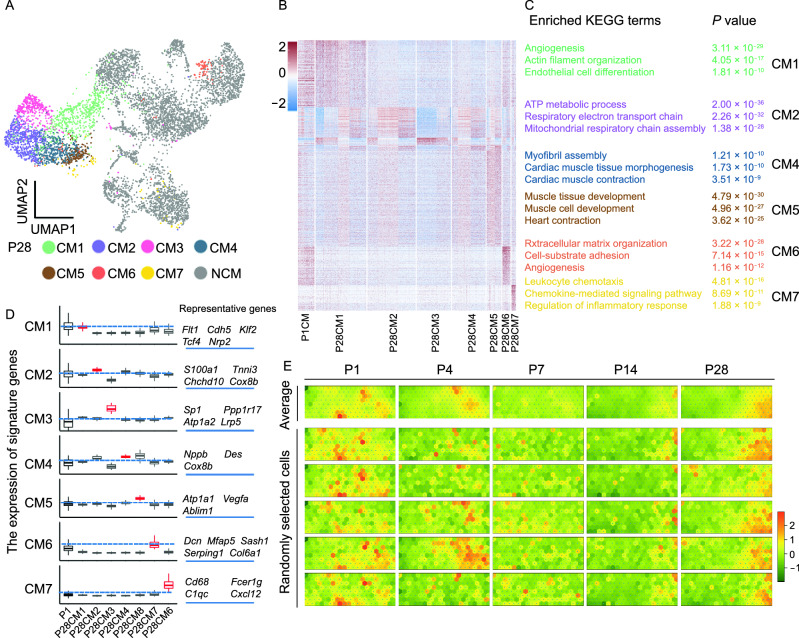
**Potential involvement of P7-CM in CM subtype specification**. (A) UMAP to show cell clustering of single cells isolated from P28 mouse hearts, *n* = 5 mice. Each dot represents a single cell. Cell populations were identified by the expression of established marker genes. Please see Table S8 for the full list. (B) Heatmap showing differentially expressed genes (DEGs) in 7 CM subtypes in the P28 heart. Please see Table S9 for the full list. (C) Potential biological processes for each CM subtype by KEGG analyses of signature genes. Enriched KEGG terms for CM3 were not shown due to too few signature genes. Selected top categories are shown. Please see Table S10 for the full list. (D) Comparisons between P1-CMs and each P28-CM subtype with respect to the expression of P28-CM subtype-specific genes. The scales of Y axis are (−1, 2) for CM1 to CM5, and (−1, 4) for CM6 and CM7. (E) Self-Organizing Map (SOM) to show the expression levels of corresponding signature genes in CMs at indicated time points. Top: average expression levels in all CMs; Bottom: expression level in 5 randomly selected cells. (F) Genome tracks showing polymerase II binding and histone modifications at *Nrp2*, *Ablim1* and *Sash1* gene loci. Potential enhancers are highlighted in orange boxes. (G) Average H3K4me1 and H3K27ac ChIP-Seq read densities in P1-CM or P7-CM at signature genes of P28-CM1, P28-CM5, and P28-CM6, respectively

This phenomenon prompted us to speculate on its biological implications with regard to postnatal heart development. Upon exposure to the postnatal environment, the heart undergoes a variety of changes, including acquiring more functionally specified cell types (Gladka et al., 2018; Litvinukova et al., 2020; Wang et al., 2020a), to achieve full adaptation. Hence, we hypothesized that the early postnatal period (P1–P7) is a key step to create a transition state of CMs (P7-CMs) to facilitate subsequent subtype specification during heart maturation. To test the hypothesis, we performed additional scRNA-seq to better characterize CM subtypes in the adult mouse heart (P28) (Fig. S6A–E). Not surprisingly, adult CMs exhibited considerable diversity with at least 7 major subtypes according to UMAP visualization (Figs. 4A and S6B; Table S8). Importantly, these CM subtypes are physically present in adult hearts, and likely functionally diverse, as indicated by Kyoto Encyclopedia of Genes and Genomes (KEGG) pathway analysis of signature genes (Figs. S6F, 4B and 4C; Tables S9 and S10). CM1, expressing CDH5 and ANKRD1, may represent a population of remodeling-associated CMs (Arimura et al., 2009; Tucker et al., 2020). CM2 and CM3 (enriched for nuclear-encoded mitochondrial genes NDUFB11, NDUFA4, COX7C and COX5B) may represent populations of high energetic state-CMs (Litvinukova et al., 2020). CM4 and CM5 are primarily heart contraction-related CMs. CM6, which is enriched for stress-response genes ANKRD116 and FHL1) may represent stress response-CMs (Kwapiszewska et al., 2008; Litvinukova et al., 2020). CM7 may be cluster of CMs under inflammatory response. Comparison of gene expression profiles between signature genes of CM subtypes in P1-CMs and P28-CM subtypes unveiled a general pattern of CM subtype specification during postnatal development: marker genes of a given CM subtype were elevated compared to P1-CMs, while, concurrently, marker genes of the remaining CM subtypes were either suppressed or less elevated (Fig. 4D). For P1-CMs to undergo such specification process, they would need to separately coordinate the up- and down-regulation of a vast array of genes for each future subtype respectively, which is conceivably highly complicated and energy-consuming. Therefore, we proposed that the transcriptional changes in CMs from P1 to P7 create a permissive state from which specification paths emanate. Consistent with this idea, P7-CMs generally showed ambiguous gene signatures compared to P1- and P28-CMs, further suggesting regression of CM identity and increase in transcriptional fluctuation to facilitate subsequent specification of CM subtypes during heart maturation (Fig. 4E).

### Perturbation of CM transition process leads to improper CM subtype specification and decreased cardiac function

**Figure 5:**
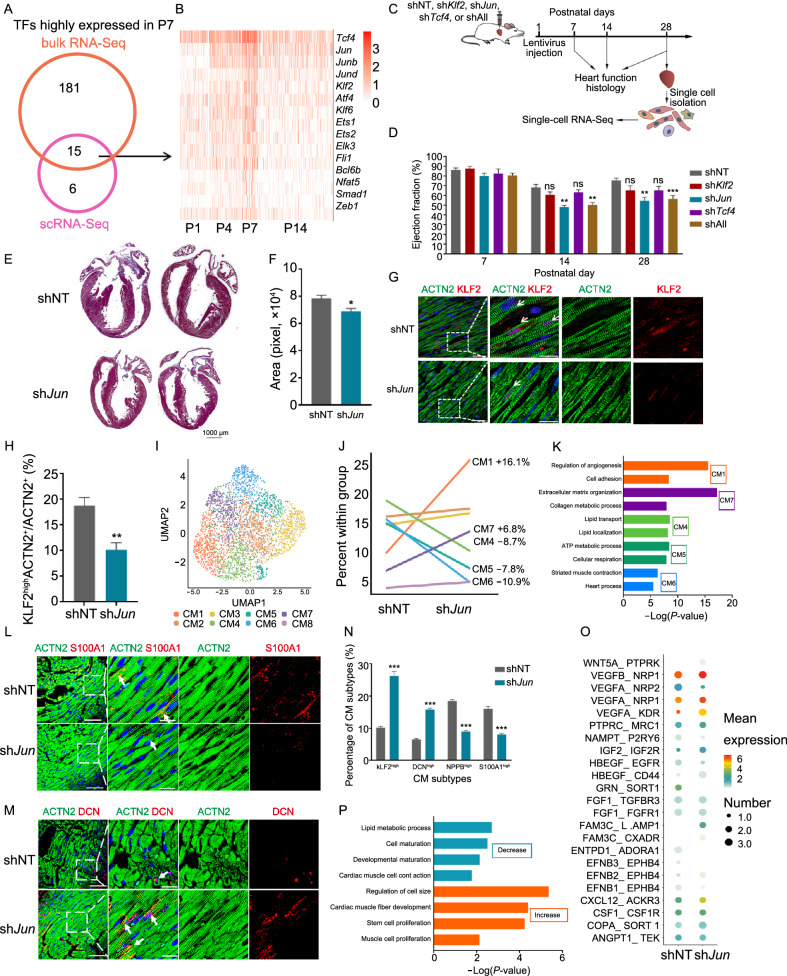
**Perturbation of CM transition process leads to improper CM subtype specification and decreased cardiac function**. (A) Overlap of TFs highly expressed in P7-CMs based on scRNA-seq and bulk RNA-seq data. (B) Heatmap to show the expression of 15 overlapping TFs in (A) at P1, P4, P7 and P14. (C) Workflow of animal experiment. Lentivirus against NT (non-targeting, shNT), *Klf2* (sh*Klf2*), *Jun* (sh*Jun*), *Tcf4* (sh*Tcf4*), or all 3 genes (shAll) were directly injected into P1-mouse myocardium, and cardiac function was evaluated by echocardiography at P7, P14, and P28, respectively. Afterwards, cells were collected from P28 hearts, and then subjected to scRNA-seq analysis. (D) Ejection fraction of hearts at different time points after lentivirus injection as measured by echocardiography. *n* = 7–12 per group, **P < 0.01, ***P < 0.001 (two-way ANOVA). (E) Masson’s trichome staining showing the histology of P28 heart sections upon NT or *Jun* silencing. (F) Quantification of (E). *n* = 3 and 7 in NT and *Jun* silencing group, respectively. *P < 0.05 (Student’s *t*-test). (G) Immunostaining of ACTN2 and KLF2 in heart sections at P7 upon NT or *Jun* silencing. White arrows indicate co-localization. Scale bar = 25 μm in lower magnification, = 10 μm in higher magnification. (H) Quantification of (G). *n* = 5 per each group, **P < 0.01 (Student’s *t*-test). (I) UMAP to display clustering of CMs isolated from hearts injected with shNT and sh*Jun* lentivirus at P28. Cells are labeled by CM subtypes. (J) Alterations of different CM subtypes in P28 hearts injected with shNT versus sh*Jun*. (K) Potential biological processes for each CM subtype by GO analyses of signature genes. Selected top categories are shown. Please see Table S11 for the full list. (L and M) Co-expression of CM subtype markers S100A1 (CM5) or KLF2 (CM1) with ACTN2 to show proportional changes of these subtypes in P28-hearts injected with shNT versus sh*Jun*. White arrows show co-localized cells. Scale bar = 25 μm in lower magnification, = 10 μm in higher magnification. (N) Quantification of immunostaining of CM subtypes. *n* = 4 per each group, ***P < 0.001 (Student’s *t*-test). (O) Alterations of ligand-receptor pairs between CMs and NCMs upon *Jun* silencing in P28 hearts. Top 20 pairs in each group are shown. Please see Table S12 for the full list. (P) GO analysis of enriched biological processes associated with decreased (blue) or increased (orange) ligand-receptors pairs. Selected top categories are shown

To fully understand biological implications of the transition state at P7, we sought to repress this process by targeting key factors. Given a predominant role of TFs in cell fate decision (Lee and Young, 2013), we first combined bulk RNA-Seq and scRNA-Seq data, and identified 15 overlapping transcription factors, including *Tcf4*, *Jun*, *Junb*, and *Jund* (Fig. 5A and 5B). Then, we screened for the functions of top-ranking TFs (*Tcf4*, *Jun* and *Klf2*) by gene silencing using lentivirus, which effectively infects proliferating neonatal cardiomyocytes, to determine their importance in CM transition state *in vivo* (Fig. 5B and 5C). *Jun* was chosen to represent transcription factor AP-1, to whom both *Junb* and *Jund* also belong. Therefore, we silenced the 3 TFs individually, or combined (shAll), in neonatal mouse hearts (P1), respectively, and evaluated their impact on heart development and cardiac function at various time points (Fig. 5C). Compared to slight changes upon *Tcf4* or *Klf2* depletion, silencing of *Jun* (sh*Jun*) or all of these 3 AP-1 components (shAll) resulted in nearly 20.12% or 18.08% decline in cardiac ejection fraction at P14, and 21.28% or 19.23% at P28, respectively (Fig. 5D). Interestingly, silencing of *Jun* also led to a 12% decrease in heart size, as characterized by Masson staining (Fig. 5E and 5F). In line with these results, *Jun* silencing resulted in a significant decrease of KLF2^high^ CMs at P7, indicating compromised P1-to-P7 transition (Figs. 5G, 5H, S7A and S7B). Together, these observations suggested a critical role of JUN in early postnatal CM transition and subsequent heart maturation.

Next, to explore the cellular basis of decreased cardiac function upon perturbation of CM transition, we performed single-cell RNA-seq on cells isolated from either shNT or sh*Jun* hearts at P28 (Figs. 5C, S7C and S7D). Major cell types were defined, and visualized by UMAP (Fig. S7E). Compared to other cardiac cell types, effective silencing of *Jun* was only evidenced in CMs, suggesting that the observed functional consequences were primarily due to JUN depletion in CMs, instead of secondary effects in other cell types (Fig. S7F). We further partitioned CMs into 8 subtypes which closed resembled 7 CM subtypes in hearts of P28 wild-type mouse (P28_CM1 to P28_CM7) (Figs. 5I, S7G and S7H). Intriguingly, compared to shNT, *Jun* silencing led to altered CM subtype proportions (Fig. 5J). For instance, proportions of clusters CM1 and CM7, which were enriched in functions related to angiogenesis and extracellular matrix organization, were increased (Fig. 5J and 5K). By contrast, proportions of CM subtypes (CM4, CM5 and CM6) with canonical CM features, such as ATP metabolic process and heart contraction, were decreased (Fig. 5J and 5K; Table S11). Consistently, altered proportions of CM subtypes upon *Jun* silencing were validated by immunostaining of sections from P28 hearts (Figs. 5L–N and S7H). Given a critical role of cell-cell interaction in the maintenance of heart function, we further took advantage of single-cell RNA-seq to evaluate the alterations of cellular interaction networks upon *Jun* silencing. By identifying differentially expressed ligands or receptors, we found dramatically changed ligand-receptor pairs between CMs and NCMs upon *Jun* silencing (Fig. 5O; Table S12). For example, Jun-depleted hearts displayed decreased ENTPD1-ADORA1 interactions, but increased interaction pairs including VEGFA-KDR, IGF2-IGF2R, etc. (Fig. 5O). Overall, increased ligand-receptor pairs upon *Jun* silencing were enriched in functions related to proliferation and development, while decreased pairs were associated with lipid metabolism, maturation and muscle cell contraction, suggesting retarded heart maturation upon *Jun* silencing (Fig. 5P). Taken together, these observations indicated that perturbation of CM transitioning may impair proper intercellular communication, which may underlie improper CM subtype specification, and thus reduced cardiac function and heart size.

### Transition state CMs isolated from P7 hearts exhibit potential in cardiac repair

**Figure 6:**
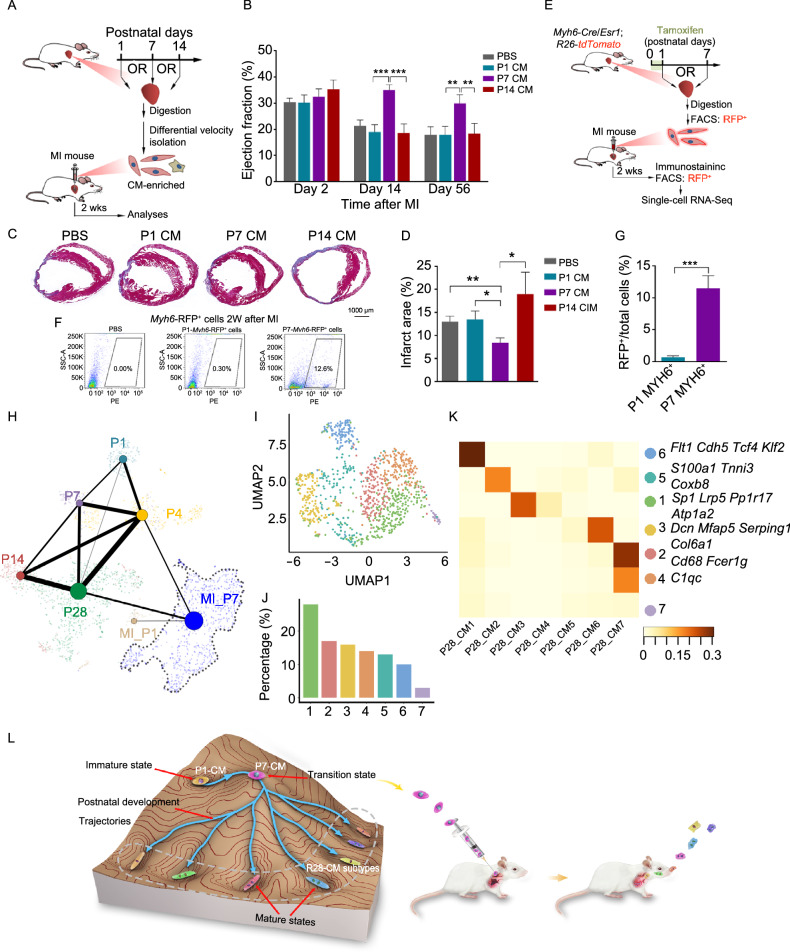
**Transition state CMs isolated from P7 hearts exhibit potential in cardiac repair**. (A) Workflow of CM transplantation into infarcted myocardium. CMs were isolated from P1, P7 and P14 hearts, respectively, and purified via differential velocity adherence (P1 and P7) or centrifugation (P14). Purified CMs were injected into infarcted hearts immediately after myocardial infarction (MI) surgery. Ejection fraction was evaluated by echocardiography 2 and 8 weeks after surgery, respectively. (B) Ejection fraction of hearts at different time points after MI as measured by echocardiography. *n* = 12–15 per group, ***P < 0.001,**P < 0.01 (two-way ANOVA). (C) Masson’s trichrome staining to show fibrosis of the heart 8 weeks upon injection of PBS, P1-, P7-, or P14-CMs after MI. Scale bar = 1 mm. (D) Quantification of (C). *n* = 6–9 per group, **P < 0.01, ***P < 0.001 (one-way ANOVA, post hoc analysis). (E) Workflow of CM transplantation into infarcted myocardium. *Myh6*-*Cre*/*Esr1*;*R26*-*tdTomato* mice were treated with tamoxifen immediately after birth. CMs were collected from P1 and P7 hearts by fluorescence-activated cell sorting (FACS), and were injected into infarcted areas immediately after MI surgery, respectively. Mice were sacrificed 2 weeks after surgery, and cardiomyocytes were harvested for scRNA-seq analysis. (F) FACS to sort for RFP^+^ cells from cell suspensions of digested heart tissues. The proportion of RFP^+^ cells are shown for each condition. (G) Quantitative analysis of RFP^+^ cells 2 weeks after injection. *n* = 5, ***P < 0.001, Student’s *t*-test. (H) Developmental trajectory of CMs from P1, P4, P7, P14, P28, MI_P1, and MI_P7 hearts. Each dot represents a cell, lines indicate possible transformation, and thickness of the line indicates probability of cell transformation between 2 stages (Please see METHODS for details). MI_P1: RFP^+^ cells collected from P1-*Myh6*-RFP^+^ cells injected hearts 2 weeks after MI; MI_P7: RFP^+^ cells collected from P7-*Myh6*-RFP^+^ cells injected hearts 2 weeks after MI (E). (I) UMAP displaying clustering of single cells from MI_P7. (J) Proportion of each cell cluster in (I). (K) Correlation analysis of cell clusters between MI_P7 (I) and CMs in P28 heart. Right: Representative marker genes of each cell cluster in MI_P7. (L) Illustration of the proposed model of postnatal heart development. At birth, fetal gene expression program is still active in neonatal CMs (P1) (immature state). During early postnatal maturation, CMs work against the stability to reach a transition state at P7, which then triggers the maturation program. P7-CMs bear the potential to differentiate into CM subtypes in the mature heart (P28). Therapeutically, transplanted P7-CMs preserve cardiac function of infarcted hearts

Given that P7-CMs exhibited features of a founder state for subsequent CM specification, we sought to determine whether they were capable of repairing damaged myocardium. To this end, we isolated and injected CMs from P1, P7 and P14 hearts into left anterior descending coronary artery ligation (LAD)-ligated, infarcted mouse hearts (Fig. 6A). Intriguingly, compared to P1-CMs and P14-CMs, P7-CMs injection resulted in markedly preserved ejection fraction at day 14 and day 56, respectively (Fig. 6B). Additionally, cardiac fibrosis was significantly alleviated after at P28 in the P7-CM injection group, as characterized by Masson’s trichrome staining (Fig. 6C and 6D). To trace the fates of injected CMs and explore the molecular basis of improved cardiac function, we isolated CMs from *Myh6-Cre*/*Esr1*;*R26*-*tdTomato* mice at P1 and P7, and injected FACS-sorted RFP^+^-cells into infarcted hearts, respectively (Fig. 6E). Two weeks following injection, isolated cardiac cells were sorted again, and RFP^+^-cells were then subjected to scRNA-seq (Fig. 6E). Noteworthily, both FACS and immunofluorescence revealed that the number of RFP^+^ cells in infarcted mouse hearts was significantly higher in hearts injected with P7 cells (12.6%) than in those injected with P1 cells (0.30%), indicating better engraftment of P7-CMs (Fig. 6F and 6G). To explore the molecular basis of superior engraftment for P7-CMs, we compared gene expression signatures of P1-CMs versus P7-CMs two weeks after injection. While P1-CMs showed molecular signatures relevant to metabolism and heart contraction, genes abundantly expressed in P7-CMs exhibited functional enrichment in cell adhesion and cytokine production, which may underlie their greater survival and engraftment (Fig. S8A and S8B). These results implied superior engraftment of P7-*Myh6*-RFP^+^ cells, which may underlie the improved cardiac outcome upon P7-CM transplantation.

Through constructing developmental trajectories with previous data, injected P7-*Myh6*-RFP^+^ cells, compared to their P1 counterparts, displayed molecular features closer to P28-CMs (Fig. 6H). A more careful inspection by cell classification revealed that injected P7-*Myh6*-RFP^+^ cells evolved into a complex mixture of 7 cell subtypes 2 weeks after injection (Fig. 6I and 6J). Interestingly, these 7 cell subtypes were reminiscent of the 7 CM subtypes in the mature heart (P28) (Fig. 6J and 6K). Taken together, these results indicated that P7-CMs possessed greater potential for CM subtype specification, and revealed transition state CMs at P7 as a potential cell source in cardiac repair after injury.

## Discussion

Compared to the huge body of studies on embryonic heart development (Wu et al., 2006; Moskowitz et al., 2007; Gupta and Poss, 2012; Xin et al., 2013; Sturzu et al., 2015; Zhang et al., 2016; Del Monte-Nieto et al., 2018; Sereti et al., 2018), much less is understood about the cellular and molecular dynamics of mammalian postnatal heart development. For example, cell fate switching is known to play a pivotal role in embryonic development (Su et al., 2018; Wagner et al., 2018). However, whether such mechanisms underlie postnatal development is hitherto unclear. By deploying bulk RNA-seq, ChIP-seq and scRNA-seq, we unexpectedly observed that cardiomyocytes at P7 exhibited robust decline of cardiac gene expression, and showed evidence of increased mRNA processing, protein synthesis and stemness. This was surprising given that heart maturation is traditionally viewed as a unidirectional process involving the progressive inactivation of fetal genes and activation of mature programs. Upon further calculations based on single-cell transcriptomes, we determined that P7-CMs served as a transition state where immature P1 CM need to pass through to achieve full maturation and CM subtype specification (Fig. 6L). This model differs drastically from the traditional view in that it demonstrates the presence of a molecularly “vague” state of CMs in the early postnatal heart that has unique functional implications. Given that we also made similar observations in additional mouse and rat strains, it is worth exploring whether this mechanism is also in play in other organs, or even in other species, such as human.

Although high-throughput approaches were employed in previous studies to dissect the molecular basis of heart development, our study still provides important new insights into this sophisticated process. In fact, as summarized in Table S13, most previous studies were performed using heart tissues, instead of isolated cardiomyocytes (O'Meara et al., 2015; Talman et al., 2018). In addition, the lack of the key time point P7 in other studies could be another explanation for their failure to capture this postnatal transition event (DeLaughter et al., 2016; Hu et al., 2018). Indeed, we performed similar bulk RNA-Seq using ventricular tissues, and failed to observe the gene expression alterations as seen using pure CMs (data not shown). These findings highlight the complexity when analyzing data from tissues with mixed cell types, and call for efforts to delineate cell behaviors at higher resolution. Even in single-cell studies, the major focus was on embryonic heart development and developmental diseases (DeLaughter et al., 2016; Hu et al., 2018). In other studies, cardiomyocyte nuclei were used in lieu of whole cells (See et al., 2017; Hu et al., 2018; Tucker et al., 2020). Lack of proper platform for large-scaled analysis of intact adult cardiomyocytes has impeded the exploration of mechanisms underlying heart maturation at higher resolution. Therefore, our study at least complements the current paradigm of postnatal heart development, and may provide insights into manipulating CM fate for therapeutic purposes. While our single-cell data suggest that this state transition applies to all CMs, it is worth noting that immunostaining results demonstrated suggested that only part of newborn cardiomyocytes (20%–40%) undergo transition. Although, using current techniques, we are unable to completely preclude possible bias introduced by digestion methods of hearts, we postulate that such discrepancy could be the result of differences in the sensitivity of detection methods, or of latency in protein changes upon RNA alterations. Thus, more sophisticated approaches are required to ascertain the degree of transition of cardiomyocytes during maturation.

Unlike fish and amphibian hearts, postnatal mammalian hearts can only regenerate injured ventricles at a very early stage (P1–P7 in mice) (Porrello et al., 2011; Notari et al., 2018). Although physiologically transient, the mechanisms underlying the regenerative potential of newborn cardiomyocyte are heavily exploited to artificially induce heart regeneration at other stages of life and under disease conditions, such as myocardial infarction (Heallen et al., 2011; Tao et al., 2016; Bassat et al., 2017; Morikawa et al., 2017; Nakada et al., 2017). Previous studies have shown that pre-existing CMs dedifferentiate immediately after injury, characterized by reduced expression of cardiac genes, and then proliferate to form new CMs to repair the injured heart muscle (Jopling et al., 2010; Kikuchi et al., 2010; Porrello et al., 2011). The CM transition process takes places between P1 and P7, coinciding with the time frame of the transient regenerative capacity of the neonatal heart, which hints at the possible participation of CM transition in heart regeneration. In addition, previous studies reported *de novo* CM formation from a pool of adult progenitor cells in the heart after injury, suggesting a potential role of resident cell sources in heart repair (Hsieh et al., 2007; Smart et al., 2011). It is therefore interesting to explore whether transition state CMs are also present in the adult heart and can be stimulated to repair damaged myocardium. Consistent with this notion, transition state cardiomyocytes at P7 displayed remarkable capacity in repairing damaged myocardium in our study, which could reflect the greater potential of P7 CMs to engraft and to specify. Thus, inducing mature CMs into a transition state may open a new avenue for heart regeneration. Intriguingly, *Jun* silencing repressed cardiomyocyte transition in our study, indicating its importance in regulating this process. Whether ectopic expression of *Jun* or other factors are capable of inducing CM transition at other stages of life warrants future investigation.

## MATERIALS AND METHODS

### Animal experiments

All studies were performed according to the guidelines of the Animal Care and Use Committee, Experimental Animal Center, Fuwai Hospital, National Center for Cardiovascular Diseases, China. Mice and rats with different genetic backgrounds and indicated ages were purchased from Beijing Vital River Laboratory. Unless otherwise specified, mice refer to the C57BL/6 strain. All mice for genetic lineage tracing analyses were purchased from the Shanghai Model Organisms Center, and were bred and housed in the same room. Mice were randomly assigned into groups by drawing lots. Echocardiographic analyses were performed by an independent investigator blinded to the procedure (myocardial infarction, MI).

#### Lineage tracing experiments

Mice with *Myh6* labeling were achieved by crossing *Myh6-cre/Esr1* mice and *R26-tdTomato*. Four micrograms of tamoxifen were orally delivered to maternal mice on postnatal days (P) 0–4 after delivery of offsprings. For cell transplantation experiments, RFP^+^ cells (mainly MYH6^+^ cells) from *Myh6-Cre*/*Esr1*;*R26*-*tdTomato* mice were sorted by a flow cytometer (BD, FACSAria2).

#### Myocardial infarction model

Wild-type male C57BL/6 mice (23**–**25 g, 8**–**10 weeks) underwent myocardial infarction surgery through permanent ligation of the left anterior descending artery (LAD). Immediately after ligation, 2 × 10^5^ of freshly isolated unlabeled cardiomyocytes or RFP^+^ (sorted by flow cytometry) cells, or 20 μL PBS vehicle control, were injected directly into the border zones surrounding infarct regions. Two weeks post injection, ejection fraction was measured by echocardiography. On the same day, mice were sacrificed, and the RFP^+^ cells were sorted by a flow cytometer (BD, FACSAria2) for scRNA-seq and hearts were collected for further analysis.

#### Genetic knockdown experiment

For gene knockdown experiments, JUN, TCF4 and KLF2 shRNA oligos were purchased from the Beijing Genomics Institute with target sequencing. The shRNAs were cloned into pLKO.1 vector according to the protocol, and knockdown efficiency of lentivirus was tested in P1 CMs *in vitro*. Then, the lentivirus was concentrated by Fast-Trap Lentivirus Purification and Concentration Kit (Millipore, FTLV00003) for mouse heart injection. Ten microliters of concentrated shNT, sh*Jun*, sh*Tcf4*, sh*Klf2*, or shAll (*Jun* + *Tcf4* + *Klf2*) lentivirus were injected into left anterior ventricles at 3 points in P1 mouse hearts, respectively. The time point of injection was chosen to allow sufficient time of viral integration, and to be sufficiently early so as to block the gradual state transition from P1 to P7. Expression of *Jun*, *Tcf4* and *Klf2* was assessed 28 days post injection. Expression of KLF2 in CMs was examined to validate inhibition of the transition state. Heart function was evaluated 28 days post injection. Afterwards, the cells from the shNT and sh*Jun* P28 mouse hearts were isolated using the Langendorff method for scRNA-seq.

### ChIP-Seq and RNA-Seq sample preparation

For isolation of cells from neonatal mouse ventricles, P1 and P7 C57BL/6 mice were sacrificed, and hearts were transferred to PBS-containing heparin sodium (1000 units in 50 mL PBS) for washing. Ventricles were chopped into small pieces, and digested with the Pierce™ Primary Cardiomyocyte Isolation Kit (Thermo, 88281) according to the manufacturer’s instructions with minor modifications. Specifically, P7 tissues were cut into relatively larger tissue chunks compared to P1, and a shorter digestion time of 20–25 min was applied. To obtain relatively pure cardiomyocytes and non-cardiomyocytes, we used the differential velocity adherence method to culture isolated cells for 1 h, and non-adherent cells were collected as cardiomyocytes. For isolation of cells from P21 and P56 mouse hearts, mice were injected with heparin sodium 100 μL (1000 units in 50 mL PBS) 20 min before sacrifice, and hearts were transferred to Tyrode’s solution for washing, after which they underwent digestion using the Langendorff perfusion technique. In brief, hearts were perfused with Tyrode’s solution for 5 min using the Langendorff apparatus, followed by digestion with the heart dissociation enzyme stock solution (0.7 mg/mL Collagenase type II and 0.7 mg/mL Bovine Serum Albumin Tyrode’s Solution) for 30 min. Tissues from ventricles were collected, chopped into pieces, and gently dissociated into single cells. Digestion was stopped by Tyrode’s solution containing 10% FBS. Finally, cell suspension was centrifuged (100 ×*g*, 2 min at room temperature) three times to separate cardiomyocytes from non-cardiomyocytes. Cardiomyocytes were collected for further experiments. Aside from C57BL/6 mice, this method was also applied to KM mice, NIH mice, Sprague Dawley rats and Wistar rats.

### Chromatin immunoprecipitation followed by high-throughput sequencing (ChIP-Seq)

ChIP for Polymerase II (Active Motif, 39397), H3K4me1 (Active Motif, 39298), H3K27ac (Active Motif, 39135), H3K4me3 (Active Motif, 39159) and H3K27me3 (Active Motif, 39155) were performed as described previously (Wang et al., 2014). Briefly, mouse ventricular cardiomyocytes were fixed using 1% formaldehyde for 10 min, followed by 0.125 M glycine for 5 min to stop the fixation. Cells were harvested, and DNA was fragmented to 300–500 bp by sonication for 8 min with a Covaris S220 sonicator. Immunoprecipitation was performed with antibodies conjugated to Dynabeads Protein G beads (Life Technologies, 1004D). ChIP DNA was eluted, reverse cross-linked, extracted with phenol/chloroform, and precipitated. For ChIP-Seq, 1 ng ChIP DNA or input DNA was used to generate sequencing libraries using the Nextera XT DNA sample preparation Kit (Illumina, FC-131-1024) according to manufacturer’s instructions. Libraries were sequenced on the NextSeq500 sequencer (Illumina) using the 35nt paired-end sequencing protocol. Two biological replicates were performed for each ChIP-Seq sample, and reads were combined for analysis.

### RNA-sequencing

Total RNA was extracted from mouse ventricular cardiomyocytes using the GeneJet RNA Purification Kit (Thermo Scientific, K0732). One μg of RNA was used for the generation of sequencing libraries using the TruSeq RNA Library Prep Kit V2 (Illumina, RS-122-2002) according to manufacturer’s instructions. All libraries were sequenced on the NextSeq500 sequencer (Illumina, FC-404-2005) using the 35 nt paired-end sequencing protocol.Two biological replicates were performed.

### Immunostaining

Immunostaining was performed according to standard protocols for paraffin embedded samples and frozen sections, respectively. For paraffin sections, hearts were collected in PBS on ice in 4% PFA at room temperature for 12–24 h depending on tissue size, followed by tissue dehydration and embedding. Embedded hearts samples were sectioned to a thickness of 4 μm. Prior to staining, sections were dewaxed, and antigen was retrieved with an EDTA buffer or citric acid solution using a pressure cooker. For frozen sections, fresh heart tissues were embedded in OCT Compound (Tissue-Tek, 4585) and then stored at −80 °C until sectioning to a thickness of 10 μm. Frozen sections were washed in water several times to remove OCT, then fixed in 4% PFA at room temperature for 10 min, and stored at −80 °C until use for immunostaining. For immunostaining, tissue sections were blocked with PBSST (0.3% Triton X-100 in goat serum working solution) for 90 min at room temperature, followed by incubation with the primary antibody overnight at 4 °C. The next day, tissue sections were washed 3 times with PBS, incubated with Alexa-Fluor-conjugated secondary antibodies for 1.5 h at room temperature, washed 3 times with PBS, and mounted with mounting medium containing the nuclear stain DAPI. The following antibodies were used (ratios indicate antibody dilution): tdTomato-RFP (Rockland, 600-401-379; 1:1000), TNNT2 (Abcam, ab8295; 1:200), ACTN2 (Abcam, ab9465; 1:200), PCM1 (Santa, sc-398365; 1:100), CDH5 (Abcam, ab91064; 1:100), KLF2 (ABclonal, A16480; 1:50), DCN (ABclonal, A1669; 1:100), CD68 (ABclonal, A15037; 1:100), NPPB (Abclonal, A2179; 1:50), S100A1 (Abclonal, A8560; 1:50), JUN (Abcam, 40766; 1:100) and ATP1A2 (ABclonal, A13850; 1:50), GAPDH (Proteintech, 10494-1-AP; 1:500). Alexa Flour488 Rabbit IgG, Alex Fluor and 594-AffiniPure Goat Anti-Mouse IgG, and Alex Fluor 647-AffiniPure Goat Anti-Rat IgG secondary antibodies (Zhongshanjinqiao, ZF-0511) were used for secondary antibody incubation. Fluorescent images were captured using a laser scanning confocal microscopy (LEICA, SP8).

Quantification of immunohistochemical staining was performed using the image analysis software ImagePro Plus. Specifically, the integrated optical density (IOD) is obtained summing all optical density values of each brown dot within an image. Mean density is calculated by dividing IOD by the area of the distribution area of the target of interest.

### Histology

Hearts were collected and fixed in 4% paraformaldehyde in PBS overnight at 4 °C and then processed for either paraffin or cryo embedding. Haematoxylin and eosin and Masson’s trichrome staining were performed according to standard procedures on paraffin sections using Automatic Dyeing Machine (Thermo Scientific Gemini AS).

### Single-cell RNA-sequencing

Single-cell RNA-Sequencing was performed using SMARTer ICELL8 Single-Cell System (Takara Bio) as previously described. In brief, single cell suspension was filtered with a 100-μm nylon cell strainer (Falcon 352350) and counted. Afterwards, cells were stained with Hoechst 33342 and Propidium Iodide (Molecular Probes**®**R37610) by adding 2 drops (~80 µL) of each dye per mL of cells, mixed carefully, and incubated at 37 °C for 20 min. Then, the reaction was stopped by adding the same volume of pre-warmed 1× PBS, and cells were pelleted by centrifugation at 100 ×*g* for 3 min at room temperature. To achieve maximum single cell collection, the cell suspension was diluted to approximately 2,200 cells/100 µL with Second Diluent 2× (WaferGen 430-000239-0001), RNase Inhibitor (Invitrogen AM2694), and pre-warmed 1× PBS (no Ca^2+^ or Mg^2+^, pH 7.4). After dilution, cells were dispensed into the ICELL8 Chip (WaferGen430-000245-0001) by the MultiSample NanoDispenser (MSND), and the chip was imaged by an imaging system. “DAPI” (to ensure single cell selection) and “Texas Red” (to exclude dead cells) were applied to select single live cells for subsequent analysis. After single cells selection, the reverse transcription (RT) reaction mix was prepared in a 220 µL total volume containing 5 mol/L Betaine Solution (Sigma B0300-1VL) 56 µL, 25 mmol/L dNTP mix (Clontech639132) 24 µL, 1 mol/L MgCl_2_ (Thermo Fisher ScientificAM9530G) 3.2 µL, 100 mmol/L DTT (Sigma43816-10ML) 8.8 µL, SMARTScribe^TM^5× First-Strand Buffer (Clontech639537) 61.9 µL, SeqAmp^TM^2× PCR Buffer (Clontech638504) 33.3 µL, 100 µmol/L RT E5 Oligo (WaferGen430-000240-0001) 4.0 µL, Amp Primer (WaferGen430-000241-0001), 100% Triton X-100 (Sigma T8787) 1.6 µL for cDNA synthesis. Reverse transcription was performed on a Chip Thermal Cycler (BIO-RAD 1861096 ), with a lid temperature of 72 °C and the following reaction program: 53.5 °C 5 s, 48.5 °C 180 s, 4.0 °C 360 s, 46.5 °C 5 s, 40.5 °C 5400 s, 51.7 °C 5 s, 48.5 °C 120 s, 38.5 °C 5 s, 39.8 °C 120 s, GOTO step 6 and 1 cycle, 73.0 °C 5 s, 69.5 °C 900 s, 98.0 °C 5 s, 96.0 °C 60 s, 100.0 °C 10 s, 99.5 °C 10 s, 60.0 °C 5 s, 63.5 °C 30 s, 68.0 °C 3 s, 67.6 °C 180 s, GOTO step 15 and 23 cycles, 72.0 °C 600 s, 4.0 °C hold. After reverse transcription, the cDNA was collected and purified using the DNA Clean & Concentrator kit (Zymo Research D4004) and Agencourt AMPure XP magnetic beads (Beckman, A63880) according to the manufacturer’s instructions. The quality of the cDNA product was analyzed by Bioanalyzer (Agilent 2100) and quantified with the Qubit® dsDNA HS Assay Kit (Invitrogen Q32851). When necessary, the cDNA product was diluted to about 0.5 ng/µL for library generation. The libraries were generated with the Nextera XT Library Preparation Kit (Illumina, 15032350) following the manufacturer's instructions, and were purified using Agencourt AMPure XP magnetic beads (Beckman, A63880). The resulting libraries were sequenced on the NextSeq500 sequencer (Illumina, FC-404-2005).

## BIOINFORMATIC ANALYSES

### Single-cell RNA-sequencing

#### Read processing and mapping

Raw reads were processed by the perl pipeline script developed by Takara Bio, which was originally described in detail by Leonard D. Goldstein, et al. (2017). In short, the main steps include: 1) Checking the validity of reads. Only read pairs whose read 1 uniquely mapped the pre-defined barcode tag (10 nt) and UMI (14 nt) were considered as valid. 2) Filtering read pairs by cutadapt (v1.8.1). The parameters were: -m 20 --trim-n --max-n 0.7 –q 20. 3) Aligning the reads to genomes of mouse, *E*. *coli*, mycoplasma, yeast, and adapter sequences by bowtie2 (v2.2.4) (Langmead and Salzberg, 2012). Contaminants were filtered by fastq screen (v0.5.1. 4). Clean reads were mapped to UCSC mm10 genome via STAR (v2.5.2b) (Dobin et al., 2013) and assigned to Ensembl genes (Zerbino et al., 2018) by featureCounts (subread-1.4.6-p1 command line) (Liao et al., 2014). Sequencing reads were further filtered and sorted by custom barcode filtering pipeline (See below).

#### Cell filtering and data normalization

Cell filtering steps were described in our earlier work (Yao et al., 2018). The number of captured transcripts per gene was inferred based on UMIs, and read pairs with UMIs containing Ns were excluded. UMIs limited to 2 s.d. from the mean of log10 UMI of all cells. Further, only cells with a minimally detected gene number of 500 were kept for downstream analysis. To remove the background signal, only genes that detected in at least 10 cells were considered as passing the threshold. In addition, reads mapped to all 37 mitochondrial genes were excluded (Table S14). Then, as illustrated by Butler et al. (2018), per-gene transcript counts were normalized across cells. Within each single cell, the UMI count of each gene was divided by total UMIs of that cell, and multiplied by size factor 10,000 to obtain a TPM-like value, which was then transformed to natural logarithm.

#### Read depth simulation

For C57BL/6 samples (P1 to P14), a total of 5,010 cells were isolated from 20 mice with an average raw read depth over 0.1 mol/L reads per cell, of which 4,231 cells passed our filtering criteria as described above (detected genes > min counts, detected genes in >10 cells**)**. For *Myh6-Cre*/*Esr1*;*R26-tdTomato* samples (P4 to P28) , a total of 13,317 cells were isolated from 9 mice, 11,168 of which passed the filtering criteria. For shNT and sh*Jun* samples, a total of 7,482 cells were collected, 7,049 of which passed the filtering criteria. To assess the effect of sequencing depth on the number of genes detected, and to learn the sequencing saturation level, we isolated different cell types from P1 samples, and calculated the number of genes with a TPM-like value > 0 in each cell. Then, a scatter plot was generated and a smooth trend line was added. As anticipated, the number of detected genes was saturated at our sequencing depth for the main cell types in P1 (data not shown).

#### Single cell clustering and annotation of cell state clusters

Clustering was conducted using the Seurat package (v2.3.4) (Butler et al., 2018). For C57BL/6 samples, to achieve an outlined description of different cell types and avoid potential batch effects among samples and experiments, we collected the cells from different postnatal time points, including P1, P4, P7, P14 and P56, used CCA in Seurat to align the experiments (chips) (Butler et al., 2018). A Seurat object was conducted for cells of each experiment, and nUMI was regressed out using the Scale function. Variable genes among cells were selected by their average expression level and dispersion: average log TPM-like value was restricted to be between 0.01 and 3, and the dispersion was set to be greater than 1. Then the MultiCCA function was used with the parameter num.cc = 20 for data alignment. We observed that the first ten canonical correlation vectors could explain the majority of the correlation strengths, and were therefore used for subsequent cell clustering. Main cell types, including cardiomyocytes (CM), endothelial cells (EC), fibroblasts (FB), granulocyte (GR), macrophages (MP), smooth muscle cell (SMC) and T cells (T), were determined according to the expression of known markers. Then the cells from certain time points were selected for downstream analysis. For *Myh6-Cre*/*Esr1*;*R26-tdTomato* samples (P4 to P28), to describe the CM cell clusters in a finer level, cells were clustered for each time point separately. First, the most variable genes among cells were selected by their average expression level and dispersion: average log TPM-like value was restricted to be between 0.01 and 3, and the dispersion was set to be greater than 1. Then, the selected genes were used for cell clustering. The FindClusters function of Seurat based on shared nearest neighbor (SNN-cliq) clustering method was utilized with default parameters (k = 30). Main cell types, including CM, EC, FB and MP, were identified by the expression of known markers. Simultaneously, sub-clusters of CMs were recognized for each time point. CM subclusters and non-CMs were visualized by UMAP. Then we collected CM subclusters from all time points of genetic lineage labeling samples, identified reporter-positive CMs, and combined them with P1-CMs to learn the developmental trajectory of CMs. For shNT and sh*Jun* samples, to avoid potential batch effect among individuals, and to retain true differences between shNT and sh*Jun* samples, we applied an iterative clustering strategy following these steps: 1) for each individual, cells were clustered in each individual as described above. The first 10 PCA components were used to do SNN clustering with a resolution value of 1. After clustering, the data were visualized by UMAP. Main cell types, including CM, EC, FB, MP, T and pericyte (PC), were determined based on the expression of known markers. 2) We collected each type of cells from all four individuals, examined the expression of *Jun* in shNT and sh*Jun* groups in each cell type, and performed a second round of dimensionality reduction and clustering for CM cells in isolation.

To address whether there were systematic batch effects in our data, we selected 107 house-keeping genes including 36 transcription elongation factor genes, 15 ribosomal genes, 19 RNA polymerase II subunit genes, and 37 tRNA genes (Table S3). Ninety-seven genes were detected in our data of C57BL/6 samples and 96 in genetic lineage labeling samples. Using the housekeeping genes, we generated a RLE (relative expression, i.e., the expression of one gene in a cell was divided by the median expression of the gene in all cells, then converted to log2) plot for each animal sample. The distribution of RLE values was very similar between samples and the median RLE value was zero in most samples. PCA analysis on these genes showed that all samples were mixed, and no obvious distinct pattern was observed. Moreover, any cluster in UMAP analysis comprising cells from a single animal sample was considered an outlier cluster, and therefore was removed from downstream analysis. From the UMAP result, all clusters contained cells from multiple animal samples. Hence, the difference among cell clusters represented true biological difference rather than systematic technical issues.

#### Identification of variable genes

As described by Butler et. al. (2018), the FindAllMarkers function was used to identify variable genes, with parameters test.use = wilcox, min.pct = 0.2, thresh.use = 0.2, only.positive = TRUE for clarifying the identity genes of each group of cells, and FALSE for identifying the DEGs between multi-groups of cells. The genes were filtered with a q value of 0.05. For block differential expression analysis, such as comparing P7CM and other cells, all other clusters were combined, and differentially expressed genes were filtered with a q value of 0.05 and average logFC of ±0.58. For differential expression analysis in a subgroup of cells, each subgroup was compared with the rest of the cells.

#### Analysis of cell Trajectory and functional gene categories

Monocle2 (v2.6.0) was used to study pseudotime trajectories of cells (Trapnell et al., 2014). The UMI matrix was used as input and variable genes detected by Seurat were used for building traces. Branches in the cell trajectory represent cells that have alternative gene expression patterns.

Diffusion maps and diffusion pseudotime were also used to study pseudotime trajectories of CMs. Firstly, the UMI matrix of CM cells in P1 to P14 samples was used as input to construct a single-cell experiment object using the SingleCellExperiment (v1.8.0) (Risso, 2019). Then Destiny (v3.0.0) was used to build a diffusion map (Angerer et al., 2016). Further, diffusion pseudotime (dpt) of each cell was accessed using the DPT function. Then, cells were plotted along their diffusion pseudotime. Slingshot (v1.4.0) (Street et al., 2018) was also applied for trajectory inference using function “getLineages”.

#### Analysis of cell heterogeneity

To further learn cell-to-cell heterogeneity in gene expression, we used the R package kohonen (v3.0.6) to construct SOM (self-organizing maps) for exhibiting the similarity relationships of genes in each transcriptome in a two-dimensional heat map, in which spatial neighborhood reflects expression pattern similarity (Kim et al., 2015). Genes with the most similar expression patterns were clustered as one set, which was shown by a hexagonal unit in SOM. Individual sets were located in the same fixed positions across all single-cell components.

To uncover the dynamics of CMs maturation, we used *I*_C_ (transition index) to quantitatively predict a tipping point as a critical transition state based on decrease of correlation between cells and concomitant increase of correlation between genes at each time point, as illustrated by Mojtahedi et al (2016). *I*_C_ is calculated as follows:

$${I}_{c}\left(t\right)=\frac{\langle \left|R\left({g}_{i},{g}_{j}\right)\right|\rangle }{\langle R\left({S}^{k},{S}^{l}\right)\rangle }$$

where 〈|*R*(*g*_i_,*g*_*j*_)|〉 is the absolute value of all Pearson’s correlation coefficients of all pairs of genes, and 〈*R*(*S*^*k*^,*S*^*l*^)〉 is the absolute value of all Pearson’s correlation coefficients of all pairs of cells.

To describe the dynamic expression pattern of TFs (transcription factors) from P1 to P28, we compared gene expression in P1CM and P28CM, and selected representative TFs of P1CM or P28CM with *P* value of < 0.05. Then the expression of each pair of genes, which was composed of one P1 TF and one P28 TF, was plotted for each time point. TF value was defined as the product of expression of P1 TF and P28 TF in each pair of genes. Then the TF values of each pair of genes at each time point were plotted to compare the activities of TFs during postnatal development.

#### Pairwise discreteness and index gene crossing

Pairwise discreteness test was used as described by Su et al. to test the discrete or continuous levels between Myh6 lineage tracing cell P1 to P28 and injected P1 and P7 cells in the MI model (Su et al., 2018). To further describe the correlation of cell sub clusters of P28 CM and injected P7 CM cells at a finer level, stringr (v1.4.0) and text2vec (v0.5.1) were used to compare the identity genes in each cell subcluster, and correlations between the cell subclusters in P28 and injected P7 were calculated as jaccard similarities by the sim2 function.

#### Analysis of cell-cell communication

Analysis of cell–cell communications was performed by comparing the representative ligand–receptor interactions between CMs and non-CM cell types (cell-cell pair) in each condition using CellphoneDB (Vento-Tormo et al., 2018). For each ligand-receptor interaction pair in each condition, the number of cell-cell pairs which were significant for the ligand-receptor interaction pair was calculated, and the mean expression of the ligand-receptor interaction pair was defined as the mean value of the average expression of each ligand-receptor interaction pair in all the significant cell-cell pairs. Then the ligand-receptor interaction pairs were ranked according to their mean expression, and the top 20 pairs were selected as representative ligand-receptor interaction pairs for conditions shNT and sh*Jun*, respectively. For each of the selected top ligand-receptor interaction pairs, the change was calculated using its mean expression in sh*Jun* minus that in shNT. A change over ± 0.4 was considered as increased/decreased ligand-receptor interaction, respectively.

### ChIP-sequencing

#### Assembly of genome tracks

Genome-wide localization of histone modifications (H3K4me1, H3K4me3, H3K27ac and H3K27me3) and RNA Polymerase II for each time point was determined via chromatin immunoprecipitation followed by high-throughput sequencing. Reads were mapped to genomes of mm10 by subread and mapped reads from biological replicates were merged for peak calling by MACS2 (v2.1.1) (Zhang et al., 2008). Broad peaks were called by MACS2 with default settings for broad peak calling (macs2 callpeak -t H3_bam -c input_bam -n name -B --SPMR –broad --outdir). All peaks were filtered by *P* value of 10^−6^. The total reads of each sample were normalized to 10 million to create UCSC files by HOMER (v4.9.1) (Heinz et al., 2010). Chromatin marks at promoters were evaluated by subtracting input from the ChIP tag centered on the TSS of each Ensembl transcript by the function of makeUCSCfile in HOMER (makeUCSCfile merged_H3_tag_directory –i merged_input_tag_directory -subtract -o merged_H3.bedGraph -fsize 5e7).

#### Analysis of chromatin marks at TSSs

ChIP values were evaluated and normalized by the function of annotatePeaks.pl in HOMER (annotatePeaks.pl mm9_GeneTSS1k.bed mm9 -size -5000,5000 -d merged_H3 merged_input -fpkm -noann> sum_H3.fpkm). Then, the input value was subtracted from the ChIP value as background signal. Since expression data were computed at the gene level, the value of each chromatin mark was defined as the mean value observed across all transcripts of a gene. Chromatin marks at TSSs in different gene categories were calculated as a median value of genes in each category (Table S2).

### Bulk RNA-sequencing

#### Identification of differentially expressed genes

For RNA-Seq analysis in mice, reads were firstly aligned to genomes of mm10 by subread (R version 1.24.2) (Liao et al., 2013). For rats, reads were aligned to genomes of rn6 by subread. Uniq reads were kept and then assigned to in-build refseq gene annotation of rsubread using featureCounts. Genes were further filtered, and only those with rpkm > 1 in two or more than two samples were kept for differential analysis, as described by Law et al. (2016). Differential analysis was conducted with limma (Ritchie et al., 2015). Genes with adjusted *P* value < 0.05 were taken as significantly differentially expressed genes, which were then clustered according to their expression patterns through the sampling series by hclust and cutree functions in R (Becker et al., 1988).

### Statistical analysis

All results are expressed as means ± SEM. Student’s *t*-test or Wilcoxon rank-sum test were used for comparison of 2 groups as indicated, and one-way ANOVA, then by post hoc analysis was used for comparison of 3 or more groups as indicated in the manuscript. *P* value < 0.05 was considered statistically significant.

## Supplementary Information

Below is the link to the electronic supplementary material.

Supplementary file1 (PDF 697 kb)
