# Supplementary material for: Postnatal state transition of cardiomyocyte as a primary step in heart maturation
Source: Protein Cell. 2022 Apr 8;13(11):842–62. doi: 10.1007/s13238-022-00908-4 (PMC9237199; doi:10.1007/s13238-022-00908-4)
Supplement: Supplementary file 1 — Supplementary file1 (PDF 697 kb) [file 13238_2022_908_MOESM1_ESM.pdf]

Supplementary Materials

Zheng Li et al.

This file contains:

Supplementary Figures 1-8

Supplementary Methods

Figure S1

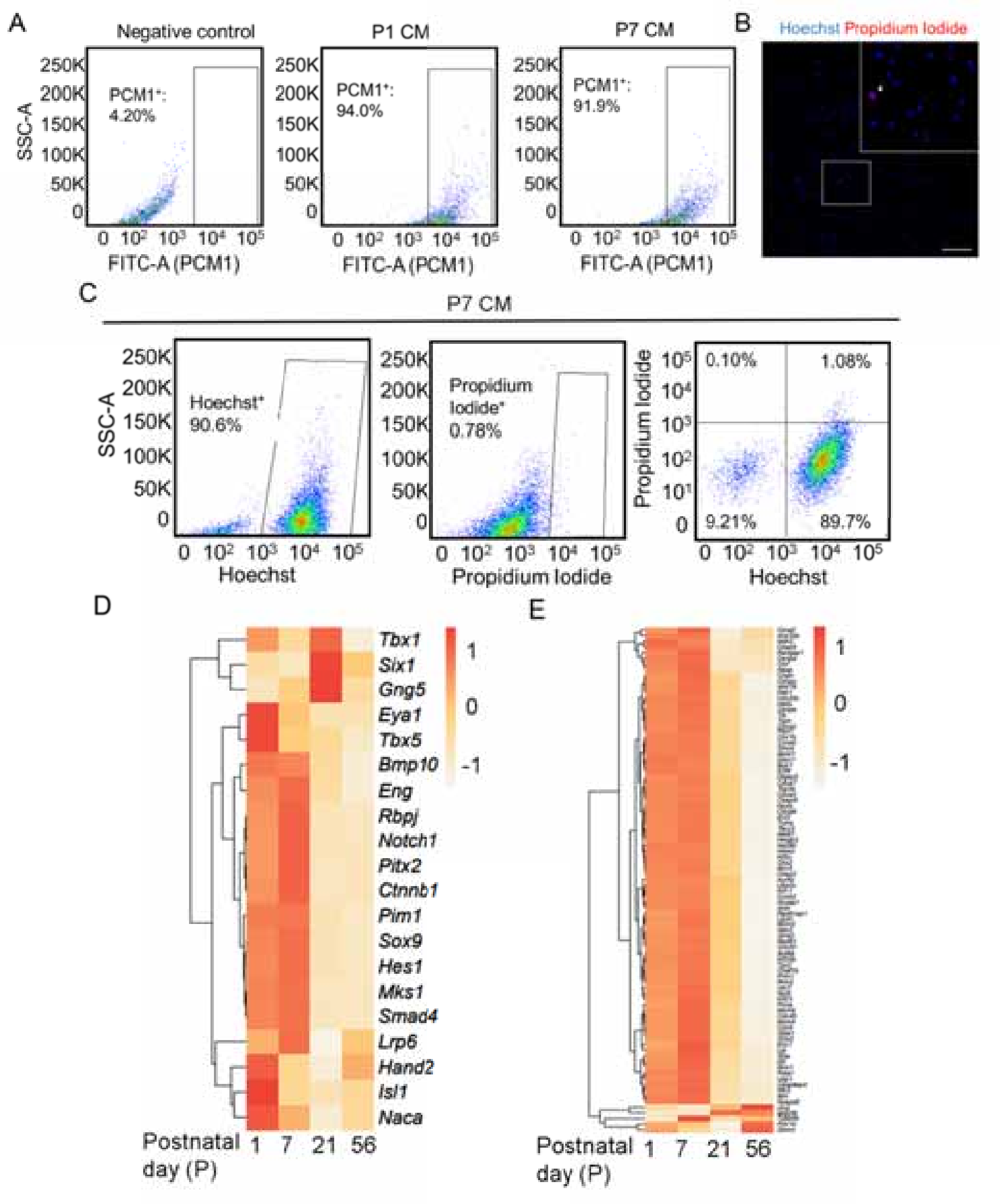

Figure S1. Quality control (QC) of isolated cardiomyocytes and heatmap of the expression of proliferation-related genes from bulk RNA-seq. (A) FACS to show P1 and P7 cardiomyocyte purity following differential velocity adherence based on PCM1 staining. **(B)** Cell viability staining of P7 cardiomyocytes using Hoechst and Propidium Iodide. The white arrow indicates the Propidium Iodide^{+} Hoechst^{+} cells (dead) cells. **(C)** FACS to show P7 cardiomyocyte viability stained by Hoechst and

Propidium Iodide. **(D)** Heatmap showing the expression of proliferation-related genes and **(E)** cell cycle related genes from bulk RNA-seq at indicated time point. **(D, E)** Heatmap showing the expression of proliferation-related genes **(D)** and cell cycle-related genes **(E)** from bulk RNA-seq data at indicated time points.

Figure S2

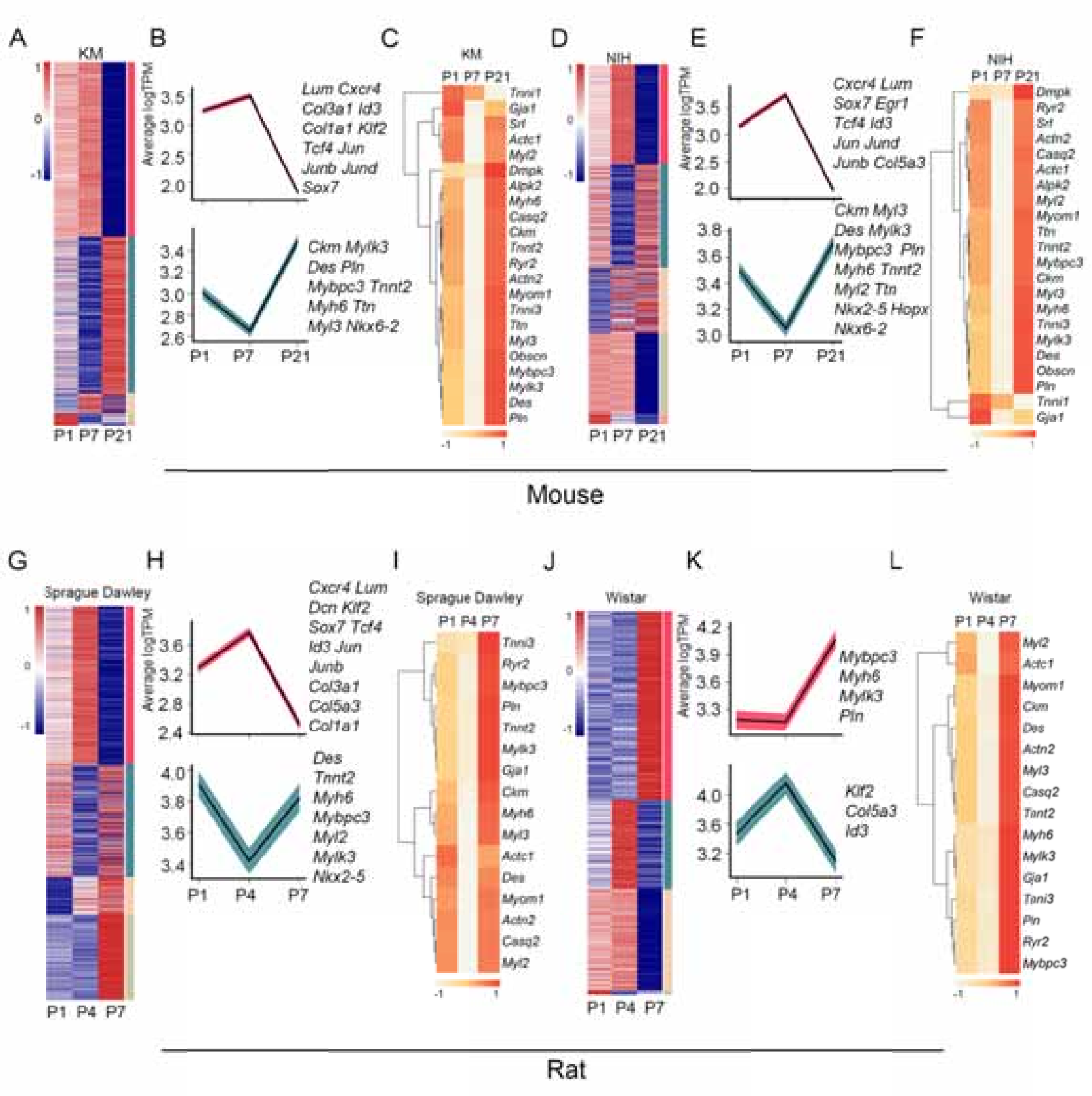

Figure S2. Transcriptomic profiling of cardiomyocytes in additional strains and species. **(A, D)** Hierarchical clustering of differentially expressed genes (DEGs) in cardiomyocytes (CMs) isolated from KM (A) and NIH (D) mice at postnatal (P) days 1, 7, and 21. **(B, E)** The mean levels (black line) and confidence interval of standard t-test (colored regions) of corresponding RNA expression for 4 clustered gene sets are shown. **(C, F)** Heatmap displaying expression of representative cardiac genes from (A) and (D), respectively. **(G, J)** Hierarchical clustering of differentially expressed genes (DEGs) in cardiomyocytes (CMs) isolated from Sprague Dawley (G) and Wistar (J) rats at P1, 4, and 7, respectively. **(H, K)** The

mean levels (black line) and confidence interval of standard t-test (colored regions) of corresponding RNA expression for 2 clustered gene sets are shown in (H) and (K), respectively. **(I, L)** Heatmap displaying expression of representative cardiac genes from (G) and (J), respectively.

Figure S3

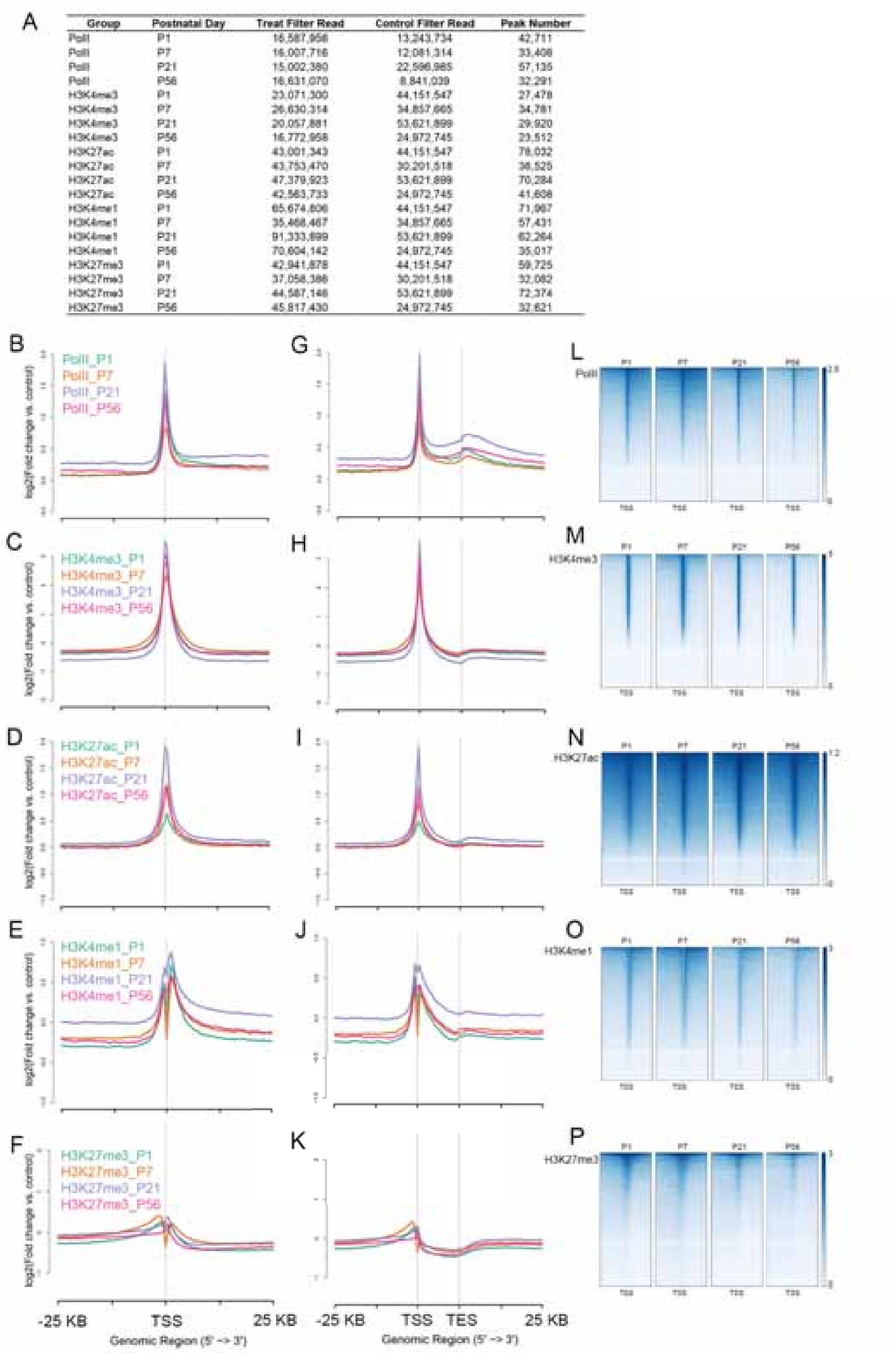

Group	Postnatal Day	Treat Filter Read	Control Filter Read	Peak Number
PolII	P1	16,587,956	13,243,734	42,711
PolII	P7	16,007,716	12,081,314	33,406
PolII	P21	15,002,380	22,596,985	57,135
PolII	P56	16,631,070	8,841,039	32,291
H3K4me3	P1	23,071,300	44,151,547	27,478
H3K4me3	P7	26,630,314	34,857,665	34,781
H3K4me3	P21	20,057,881	53,621,899	29,920
H3K4me3	P56	16,772,958	24,972,745	23,512
H3K27ac	P1	43,001,343	44,151,547	78,032
H3K27ac	P7	43,753,470	30,201,518	38,525
H3K27ac	P21	47,379,923	53,621,899	70,284
H3K27ac	P56	42,563,733	24,972,745	41,606
H3K4me1	P1	65,674,806	44,151,547	71,967
H3K4me1	P7	35,468,467	34,857,665	57,431
H3K4me1	P21	91,333,899	53,621,899	62,264
H3K4me1	P56	70,604,142	24,972,745	35,017
H3K27me3	P1	42,941,878	44,151,547	59,725
H3K27me3	P7	37,058,386	30,201,518	32,082
H3K27me3	P21	44,587,146	53,621,899	72,374
H3K27me3	P56	45,817,430	24,972,745	32,621

Figure S3. Epigenetic decline of cardiac gene expression in CMs at P7. (A) Quality control of ChIP-Seq, including read number and peak number for each ChIP-Seq at indicated time points. **(B-F)** ChIP-Seq signal distribution of Polymerase Ⅱ (B), H3K4me3 (C),

H3K27ac (D), H3K4me1 (E), and H3K27me3 (F) around transcription start site (TSS) at indicated time points. **(G-K)** ChIP-Seq signal distribution of Polymerase Ⅱ (G), H3K4me3 (H), H3K27ac (I), H3K4me1 (J), and H3K27me3 (K) across the gene bodies at indicated time points. **(L-P)** Heatmap to show (G-K).

Figure S4

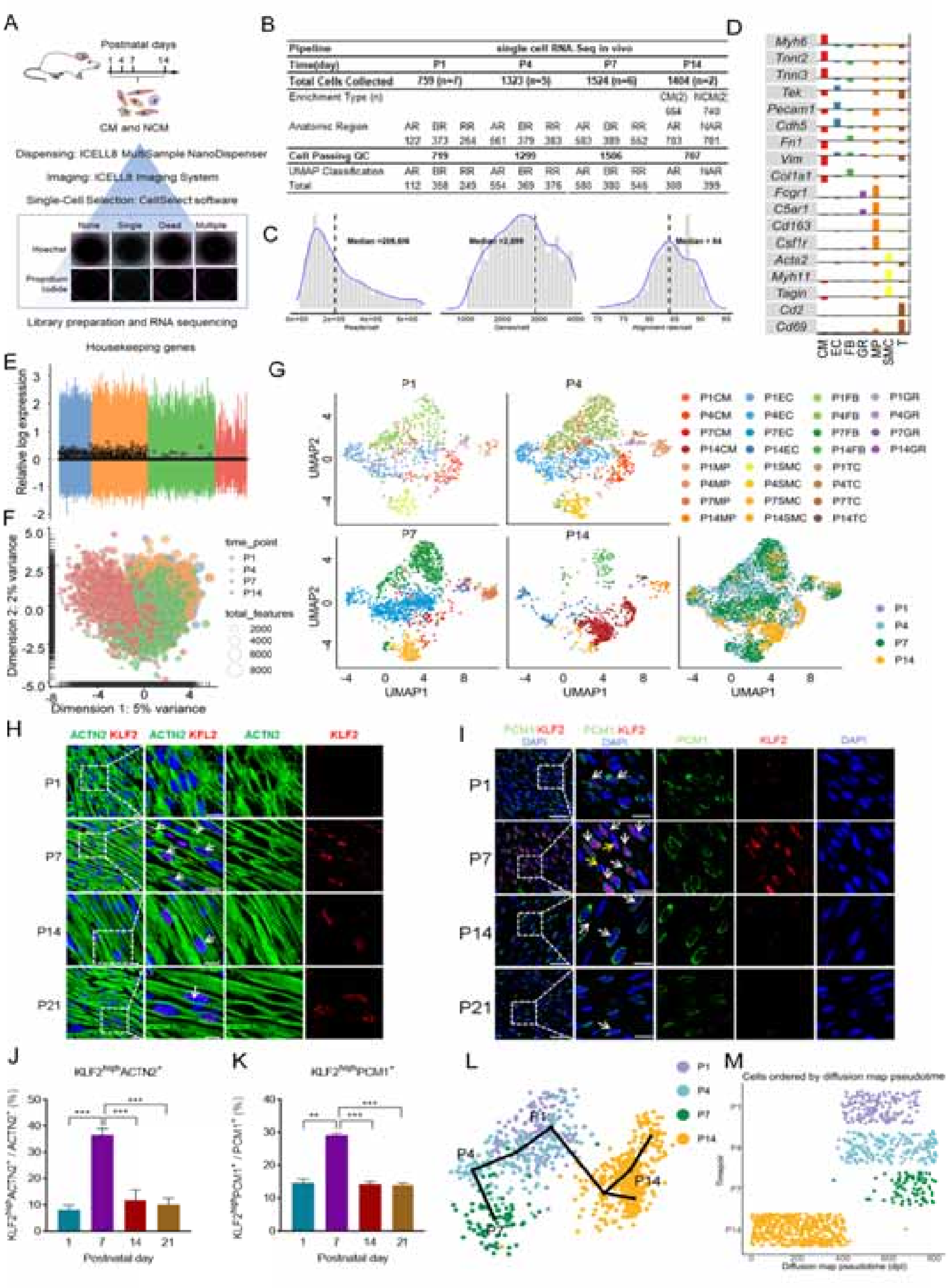

Figure S4. Workflow and quality control of scRNA-seq, and validation of P7-tCMs. (A) Schematic of study design, and workflow of Nanogrid single-cell selection and sequencing platform. Digested cells captured from different anatomic regions were stained with Hoechst and Propidium iodide (PI) before selection. Live, single cells (Hoechst+; PI-) were identified

by the Nanowell imaging system, and were selected for subsequent experiments (see Methods). **(B)** Animal information and detailed information of cells processed via scRNA-seq. AR, apex region; BR, border region of the apex; RR, remote region; QC, quality control. **(C)** Quality metrics for single-cell RNA-Seq data showing distributions of number of reads per cell, alignment rate per cell, and number of genes detected per cell. **(D)** The expression of representative marker genes in different cell types. **(E)** Relative log expression (RLE) plot of 97 housekeeping genes in each single cell captured from different mice at different time points. Each bar corresponds to the boxplot of RLE value of one cell, with a blank circle representing the median of the RLE value. Middle grey line represents interquartile range (IQR), and the extension line is the 1.5×IQR. **(F)** Principal component analysis (PCA) of all collected cells using 97 housekeeping genes. Please see Table S3 for the full list. (G) UMAP clustering of single cells isolated from hearts at indicated developmental stages CM, cardiomyocyte; EC, endothelial cell; FB, fibroblast; GR, granulocyte; MP, macrophage; SMC, smooth muscle cell; T, T cell. **(H)** Immunofluorescence staining against ACTN2 and KLF2 in heart sections at indicated time points. White arrows indicate co-localized cells. Scale bar = 25 μm in lower magnification, = 10 μm in higher magnification. **(I)** Immunofluorescence staining against KLF2 and PCM1 in heart sections at indicated time points. White arrows indicate co-localized cells. Scale bar = 25 μm in lower magnification, = 10 μm in higher magnification. **(J)** Quantification of (G). n = 6, ***P<0.001 (one-way ANOVA, post hoc analysis). **(K)** Quantification of (I). n = 6, ***P<0.001 (one-way ANOVA, post hoc analysis). **(L-M)** Slingshot (L) and Diffusion map (M) analysis showing the ordering of CMs along pseudotime. Each dot represents a single CM, while each color indicates a time point.

Figure S5

A

Pipeline	Single-cell RNA-Seq of *Myh6-Cre/Esr1;R26-tdTomato* mouse								
Time(day)	P4		P7		P14		P28		
Total Cells Collected	2705 (n=2)		2637 (n=2)		2687 (n=2)		5288 (n=3)		
Individuals	mouse1	mouse2	mouse1	mouse2	mouse1	mouse2	mouse1	mouse2	mouse3
	1233	1472	1302	1335	1438	1249	1370	1434	2484
Cell Passing QC	2375		2431		1709		4653		
Individuals	mouse1	mouse2	mouse1	mouse2	mouse1	mouse2	mouse1	mouse2	mouse3
Total	1101	1274	1198	1233	1195	514	1264	1284	2105

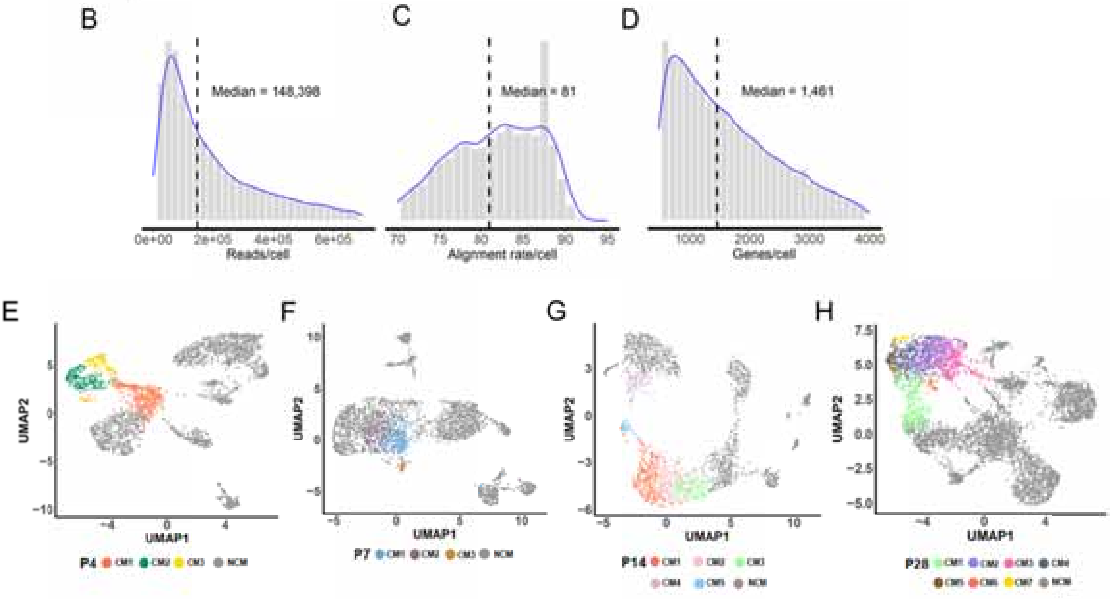

Figure S5. Animal information and quality control for scRNA-seq data of genetic lineage tracing mice. (A) Animal information and numbers of cells of *Myh6-CreER;Rs6-tdTomato* mice processed via single-cell RNA sequencing. **(B-D)** Quality metrics for scRNA-seq data showing distributions of number of reads per cell (B), alignment rate per cell (C), and number of genes detected per cell (D). **(E-H)** UMAP clustering of single cells isolated from P4 (E), P7 (F), P14 (G), and P28 (H) mouse hearts. Please see Table S7 for the full list.

Figure S6

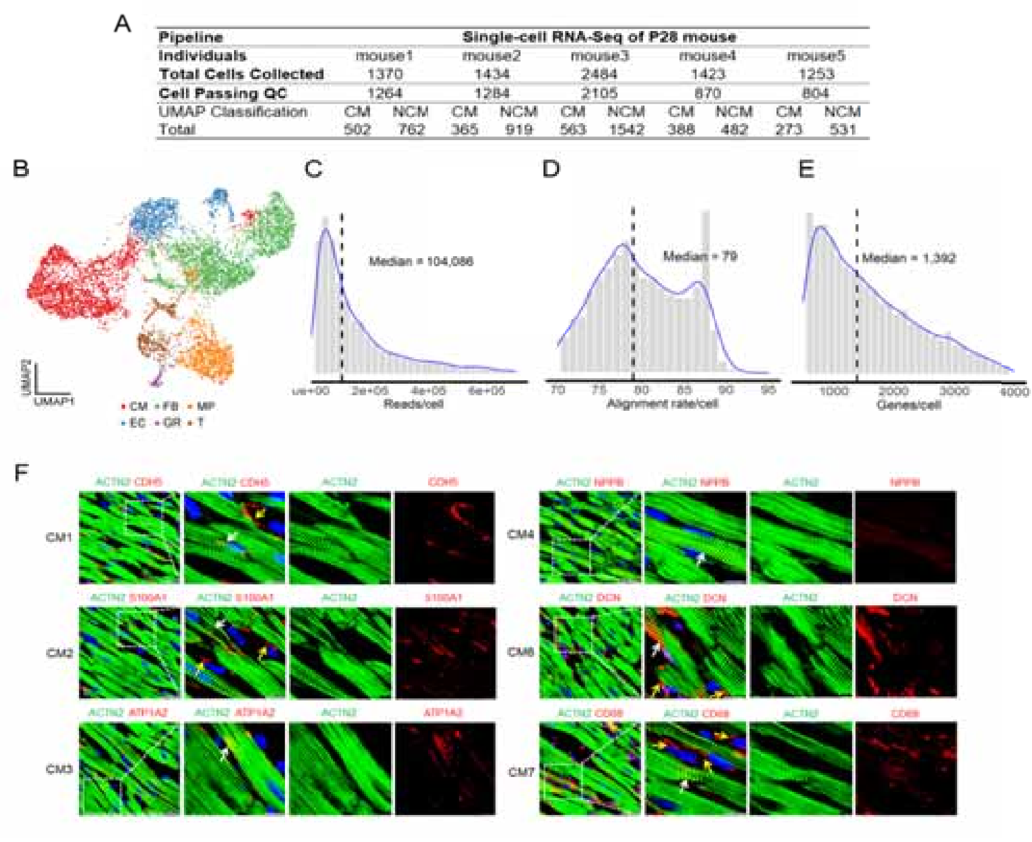

Figure S6. scRNA-seq quality control and subtype validation of P28 cardiomyocytes. (A) Animal information and numbers of cells of P28 mice processed via scRNA-seq. **(B)** UMAP clustering of single cells isolated from P28 mouse hearts. Each dot represents a single cell. Cell populations were identified by the expression of established marker genes. CM, cardiomyocyte; EC, endothelial cell; FB, fibroblast; MP, macrophage; TC, T cell; GR, granulocyte. Please see Table S8 for the full list. **(C-E)** Quality metrics for single-cell RNA-Seq data showing distributions of number of reads per cell (C), alignment rate per cell (D), and number of genes detected per cell (E). **(F)** Immunofluorescent staining of ACTN2 and different CM subtypes markers in P28 heart sections White arrows indicate co-localized cells, yellow arrows represent cells with subtype-specific marker staining only. Scale bar = 25 μm in lower magnification, = 10 μm in higher magnification.

Figure S7

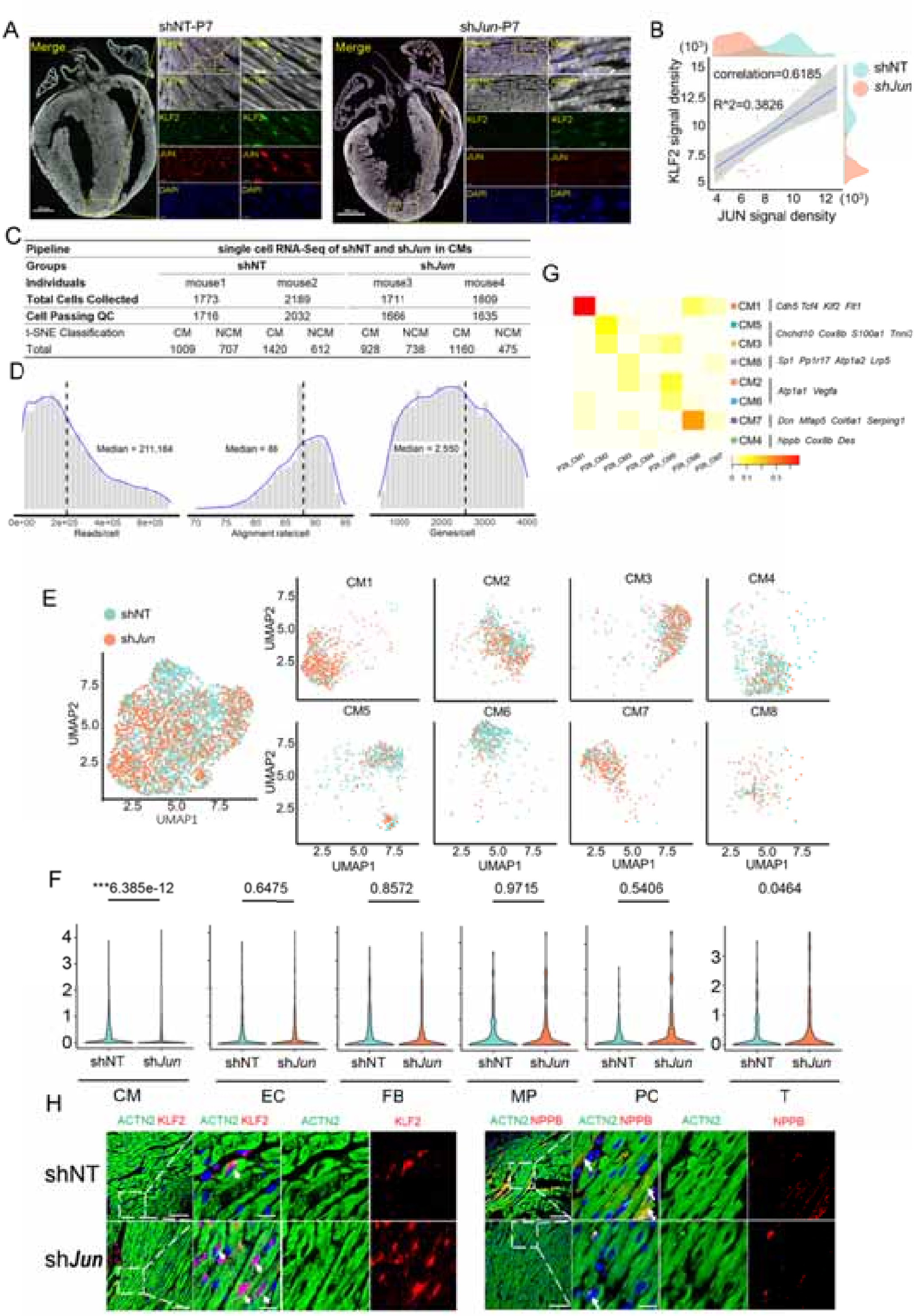

Figure S7. Quality control of Jun knockdown and its effect on CM subtype specification.

(A) Multicolor staining of KLF2, JUN, ACTN2 and DAPI of whole heart tissue after knockdown of *Jun* at P7. Yellow arrow indicated the KLF2+ JUN+ DAPI +and ACTN2+ cells.

Scale bar = 500 µm in the shNT whole heart, Scale bar = 800 µm in the shJun whole heart, Scale bar = 40 µm in lower magnification, = Scale bar 10 µm in higher magnification. **(B)** Quantitative correlation analysis of JUN and KLF2 signal density in individual cardiomyocytes of shNT and sh*Jun* groups in (A). n = 5 hearts per group, and 3 regions per section for quantitative analysis. **(C)** Animal information and numbers of cells of P28-mice processed via scRNA-seq. **(D)** Quality metrics for scRNA-seq data showing distributions of number of reads per cell, alignment rate per cell, and number of genes detected per cell. **(E)** UMAP plots showing all CMs combined (left) or each CM subtype (right) in shNT versus sh*Jun* group. **(F)** Violin plots to show the expression of *Jun* in different cell types (EC, endothelial cell; FB, fibroblast; MP, macrophage; PC, pericyte; T, T cell) of P28-hearts with shNT or shJun injection based on single-cell RNA-Seq. **(G)** Correlation analysis of CM subtypes between (5J) and CMs in P28 heart in wild type mouse. **(H)** Co-expression of CM subtype markers NPPB (CM4) or DCN (CM7) with ACTN2 to show proportional changes of these subtypes in P28-hearts injected with shNT versus sh*Jun*. Scale bar = 25 µm in lower magnification, = 10 µm in higher magnification.

Figure S8

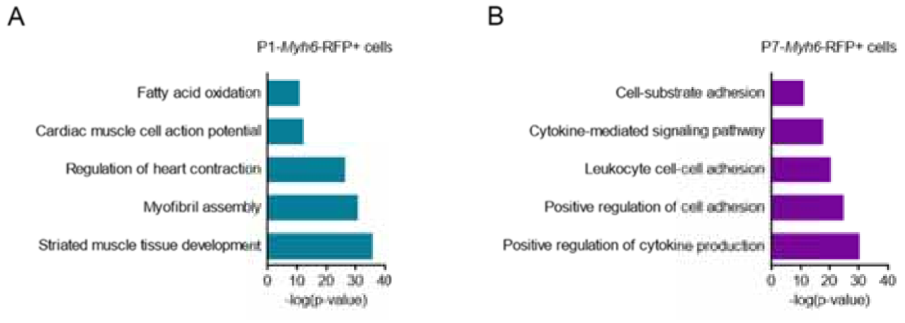

Figure S8. Transition state-CMs (tCMs) isolated from P7 hearts exhibit potential in cardiac repair. (A-B) Gene ontology (GO) analysis on genes highly expressed in P1-CMs (A) or P7-CMs (B) 2 weeks after injection based on single-cell analysis data. Selected top categories are shown here.

Supplementary methods

Multicolor immunostaining. Multicolor immunostaining was performed according to standard protocols for the multicolor kit (PANOVUE, TSA-RM-827258, 10217100100) for paraffin embedded sample sections. Briefly, hearts were collected in PBS on ice in 4% PFA at room temperature for 12-24 h depending on tissue size, followed by tissue dehydration and embedding. Embedded hearts samples were sectioned to a thickness of 4 μm. Prior to staining, sections were dewaxed, and antigen was retrieved with EDTA buffer (pH 9.0) using a pressure cooker following peroxide blocking. Then, sections were blocked using block solution, followed by incubation with the first primary antibody overnight at 4 °C. The next day, sections were incubated with the secondary antibody at room temperature for 1 hour. Repeated rounds of antigen retrieval, blocking, incubation with the next primary and secondary antibodies were repeated until sections were labeled with all three target antibodies. Finally, sections were mounted with DAPI, and subjected to multicolor scanning using Vectra Polaris System (Perkin Polaris).

Validation of cardiomyocytes purity by FACS. Isolated cardiomyocytes were fixed in 0.01% formaldehyde for 10-15 minutes, and then treated with pre-cooled methanol for 30 minutes to permeabilize the plasma membrane. Samples were incubated with an anti- PCM1 antibody incubating overnight at 4 °C, then with a secondary antibody at room temperature for 1 hour. Resulting samples were evaluated for PCM1 positivity using FACS Aria 2 (BD Biosciences). Cells without primary antibody incubation served as a negative control.

Cell viability assay. Isolated cells were incubated with Hoechst 33342 and Propidium Iodide (NucBlue® Live reagent, Thermo) for 30 minutes at 37 °C. Images were taken on a fluorescence microscope (Zeiss, Axio Observer D1) for visualizing staining. Samples were also subjected to FACS analysis to quantify Hoechst^{+}PI^{-} (live) versus Hoechst^{+}PI^{+}(dead) cells (FACS Aria 2, BD Biosciences).
